# What is the global burden of visual impairment?

**DOI:** 10.1186/1741-7015-4-6

**Published:** 2006-03-16

**Authors:** Lalit Dandona, Rakhi Dandona

**Affiliations:** 1 Health Studies Area, Centre for Human Development, Administrative Staff College of India, Hyderabad, India

## Abstract

**Background:**

A recent estimate by the World Health Organization (WHO) suggests that 161 million persons worldwide have visual impairment, including 37 million blind (best-corrected visual acuity less than 3/60 in the better eye) and 124 million with visual impairment less severe than blindness (best-corrected acuity less than 6/18 to 3/60 in the better eye). This estimate is quoted widely, but because it is based on definitions using best-corrected visual acuity, uncorrected refractive error as a cause of visual impairment is excluded.

**Methods:**

We reviewed data from population-based surveys of visual impairment worldwide published 1996 onwards that included presenting visual acuity, and estimated the proportion of visual impairment caused by uncorrected refractive error in different sub-regions of the world. We then extrapolated these data to estimate the worldwide burden of visual impairment including that caused by uncorrected refractive error.

**Results:**

The total number of persons with visual impairment worldwide, including that due to uncorrected refractive error, was estimated as 259 million, 61% higher than the commonly quoted WHO estimate. This includes 42 million persons with blindness defined as presenting visual acuity less than 3/60 in the better eye, and 217 million persons with less severe visual impairment level defined as presenting visual acuity less than 6/18 to 3/60 in the better eye, 14% and 75% higher, respectively, than the WHO estimates based on best-corrected visual acuity. Sensitivity analysis, taking into account the uncertainty of the proportion of visual impairment caused by refractive error, revealed that the number of persons in the world with visual impairment due to uncorrected refractive error could range from 82 to 117 million.

**Conclusion:**

The actual burden of visual impairment worldwide, including that caused by uncorrected refractive error, is substantially higher than the commonly quoted WHO estimate that is based on best-corrected visual acuity. We suggest that the indicative estimate of 259 million persons with visual impairment worldwide, which includes 42 million blind with visual acuity less than 3/60 in the better eye, be used for further planning of the VISION 2020 initiative instead of the often quoted 161 million estimate that includes 37 million blind.

## Background

The World Health Organization (WHO) recently completed an impressive global review of a large number of surveys on visual impairment, and estimated that there were 161 million persons worldwide with visual impairment in the year 2002, including 37 million with blindness [1,2]. This estimate is now commonly quoted, including by VISION 2020 – The Right to Sight, the global initiative launched jointly by the WHO and the International Agency for the Prevention of Blindness, which aims to help eliminate avoidable blindness globally by the year 2020 [3]. This estimate was based on the definitions of visual impairment in the International Statistical Classification of Diseases (ICD), which define blindness as best-corrected visual acuity less than 3/60 or central visual field no greater than 10 degrees in the better eye, and low vision (visual impairment less severe than blindness) as best-corrected visual acuity less than 6/18 to 3/60 [4]. These definitions of visual impairment using best-corrected visual acuity exclude uncorrected refractive error as a cause of visual impairment, thereby leading to underestimation of the total burden of visual impairment [5,6]. We therefore attempted to estimate the global burden of visual impairment, including that caused by uncorrected refractive error, by reviewing data from published population-based surveys of visual impairment that included presenting visual acuity.

## Methods

The WHO estimate of visual impairment in the different Global Burden of Disease (GBD) sub-regions, classified according to the GBD 2000 Project [7], for the year 2002 was used as the base estimate of visual impairment due to causes other than uncorrected refractive error, as best-corrected vision was used for this estimate [1]. To assess the additional contribution of uncorrected refractive error to global visual impairment from published population-based surveys, we followed the guidelines for reporting meta-analysis suggested by the Meta-analysis of Observational Studies in Epidemiology Group [8].

The PubMed literature database [9] was searched in early August 2005 and again in early December 2005. The terms "blindness AND population-based survey", "blindness AND population-based study", "visual impairment AND population-based survey", "visual impairment AND population-based study", "low vision AND population-based survey" and "low vision AND population-based study" were used to locate papers on population-based surveys of visual impairment in any language published 1996 onwards, covering about a decade up to the present. This search yielded 271 publications. The abstract of each of these publications was reviewed, and the papers that were actually population-based surveys of visual impairment and documented its causes were obtained from the journals and through contact with authors if these were in the English language. If additional papers on population-based surveys of visual impairment and its causes published 1996 onwards were found in the references of these papers located through the PubMed search, these papers were also obtained, resulting in a total of 283 publications for review. The five papers from the PubMed search that were in a language other than English were assessed on the basis of their English abstracts. The aim of this literature search was to locate publications that included presenting visual acuity to define visual impairment, which would allow us to ascertain the contribution of uncorrected refractive error to visual impairment in one or both of the categories used by WHO for reporting, i.e. visual acuity less than 3/60 in the better eye and visual acuity less than 6/18 to 3/60 in the better eye. 

The following exclusion criteria were applied while assessing the papers on population-based surveys of visual impairment:

1. Survey was only on children or only on persons 60 or more years of age, which would not allow estimates for the bulk of the adult population.

2. Definitions of visual impairment did not include either of the two visual acuity categories used by WHO for reporting, and these categories could not be derived from the categories shown.

3. Absence of clear number of persons who were visually impaired due to uncorrected refractive error or percentages of visual impairment due to uncorrected refractive error in either of the two visual acuity categories used by WHO.

4. Substantial discrepancy between the numbers/percentages mentioned in the text and tables or figures, making it impossible to determine the exact proportion of visual impairment caused by uncorrected refractive error in either of the two visual acuity categories used by WHO.

5. Participating sample size less than 1000 in the survey.

6. Data collected before 1991, which would make it too old.

7. Uncertain generalisability of data due to unspecified participation rate, i.e. the number of eligible sampled persons was not given.

8. Data from an atypical area or on an atypical population that could not be generalised to the GDB sub-region, e.g. data from an onchocerciasis endemic zone that could not be generalised to one of the African sub-regions.

9. If more than one survey were available from a country, and if at least one included all age groups, those with only adult age groups were excluded, unless the one with all age groups was on a population that was not easily generalisable.

These criteria were used to assign reasons for including or excluding each of the 283 publications from our analysis [see Additional file 1]. Only nine population-based surveys from eight countries in seven GBD sub-regions met the inclusion criteria that enabled an assessment of the proportion of visual impairment due to refractive error in one or both of the visual impairment categories used by WHO [10-19]. These seven GBD sub-regions represented 3999 million (64.4%) of the world's 6213 million population in 2002 (Table 1) [20].

If one or more qualifying surveys were available for a GBD sub-region, the data from those were utilised to arrive at the best estimates for the proportion of blindness (presenting visual acuity less than 3/60 in the better eye) and less severe visual impairment (presenting visual acuity less than 6/18 to 3/60 in the better eye) caused by uncorrected refractive error. For the GBD sub-regions for which no qualifying survey was available, the most closely matching sub-region based on mortality strata as classified by GBD [7] was selected for which a qualifying survey was available. Data on the proportional contribution of uncorrected refractive error to blindness and less severe visual impairment from this matching selected sub-region were extrapolated to the sub-region for which no qualifying survey was available. This extrapolation either used data from the matching sub-region directly, or introduced adjustments if the population characteristics were somewhat different, as explained in the results section below.

The WHO base estimates for blindness and less severe visual impairment were then adjusted by adding the contribution of uncorrected refractive error, to arrive at estimates indicating the total number of persons with blindness and less severe visual impairment including that caused by uncorrected refractive error. As clear data regarding the contribution of uncorrected refractive error to blindness and less severe visual impairment were available from only a small number of countries, and extrapolations were used to arrive at the best estimates, sensitivity analysis was done to arrive at plausible ranges for the overall estimates. This was done by assuming that the actual contribution of uncorrected refractive error could be less or more than the proportional contribution of uncorrected refractive error to blindness and less severe visual impairment estimated from the limited available data.

## Results

The data available from qualifying surveys on the contribution of uncorrected refractive error to blindness and less severe visual impairment are shown in Table 2. Use of these data, and extrapolations to sub-regions for which qualifying surveys were not available, to estimate the total number of persons with blindness and less severe visual impairment including that caused by uncorrected refractive error are shown in Table 3. The total number of persons with visual impairment worldwide, including that due to uncorrected refractive error, was estimated as 259 million, 61% higher than the WHO estimate based on best-corrected visual acuity definition. This includes 42 million persons with blindness and 217 million persons with less severe visual impairment, 14% and 75% higher respectively than the WHO estimate based on best-corrected visual acuity. Of the total blindness worldwide, 12.3% was estimated to be due to uncorrected refractive error; and of the total less severe visual impairment, 42.8% was estimated to be due to uncorrected refractive error. Of the 98 million persons worldwide estimated to be visually impaired because of uncorrected refractive error, the highest numbers were in the Western Pacific region including China (28 million) and the South-East Asia region including India (25 million).

Sensitivity analysis was done assuming that the proportional contribution of uncorrected refractive error to blindness could be 20% less or more than our estimate of 12.3% (i.e. 9.8% to 14.7%), and that the corresponding contribution to less severe visual impairment could be 10% less or more than our estimate of 42.8% (i.e. 38.5% to 47.1%). A higher percentage variation in the plausible range for blindness was assumed, as the proportional contribution of uncorrected refractive error to blindness was smaller than that for less severe visual impairment, and smaller proportions may be associated with larger percentage variations. This sensitivity analysis revealed that the total number of persons worldwide who were visually impaired because of uncorrected refractive error could range from 82 million (4 million blind and 78 million with less severe visual impairment) to 117 million (6 million blind and 111 million with less severe visual impairment).

## Discussion

The obvious limitation of our estimates for the contributions of uncorrected refractive error to blindness and less severe visual impairment is the sparseness of relevant published data from around the world. Although we did an extensive PubMed search and examined cross-references from the papers located, we did not search non-English-language databases, which might have led us to overlook some relevant papers. Faced with the scanty data from the literature search, we had the option of aborting this exercise or making the best estimates using the most reasonable assumptions. As estimates of visual impairment worldwide without inclusion of uncorrected refractive error are obviously underestimates, and because frequent reference to such underestimates is not only inaccurate but can be inadvertently misleading, we felt that it was better to make initial indicative estimates of visual impairment worldwide including uncorrected error with whatever published data were available.

Our indicative estimates suggest that the number of visually impaired persons in the world is about 259 million, including 42 million blind with presenting visual acuity less than 3/60 in the better eye. This estimate includes 98 million persons with visual impairment due to uncorrected refractive error, who are not included in the WHO estimate of 161 million visually impaired persons worldwide based on the ICD visual impairment definitions that use best-corrected visual acuity [1]. Because the contribution of uncorrected refractive error to visual impairment was uncertain due to the limited available data, we estimated the plausible range of the number of persons worldwide who were visually impaired due to uncorrected refractive error as 82 to 117 million. The original WHO estimate of visual impairment due to causes other than uncorrected refractive error does not report a plausible range because of the uncertainty that may be associated with the available data [1]. Ideally, all worldwide estimates of visual impairment should include a plausible range based on the degree of uncertainty of the data, as is done by UNAIDS for HIV estimates [26].

We estimated from the available data that the proportional contribution of uncorrected refractive error to visual impairment was highest in the GBD sub-region that includes China, and lowest in the GBD sub-regions of Africa. This is consistent with the highest rates of refractive error reported for Chinese populations [23-25], and a review suggesting a relatively low contribution of uncorrected refractive error to blindness in Africa [27].

We have used the generic term 'uncorrected refractive error' in this paper to cover both refractive error that is not corrected at all and that which is inadequately corrected if a person is using refractive correction but is still visually impaired. It is important to point this out, as several studies from different parts of the world have suggested that in addition to completely uncorrected refractive error, inadequately corrected or under-corrected refractive error is also a significant problem [28-33].

It is important to note that refractive error is the most easily treatable cause of visual impairment, in most cases by simple spectacles. In addition, since the onset of visual impairment due to natural refractive error sets in at a younger age than the other major causes, it is responsible for a much larger number of blind years lived by a person than most other causes if left uncorrected [5,14]. It was estimated in an Indian state that blindness due to uncorrected natural refractive errors resulted on average in over 30 years of blindness for each person as compared with 5 years of blindness due to untreated cataract for each person [14]. Not only do our estimates indicate that uncorrected refractive error is the most common cause of visual impairment in the world, the burden it causes in the more productive younger years of life has a potentially serious adverse socio-economic impact on society. This underscores the point that visual impairment due to uncorrected refractive error cannot be overlooked in worldwide estimates, even though the currently available data regarding it are not extensive. Our indicative estimates can obviously be refined as more data become available from around the world. For now, we suggest that the indicative estimate of 259 million persons with visual impairment worldwide, which includes 42 million blind with presenting visual acuity less than 3/60 in the better eye, is more appropriate for further planning of the VISION 2020 initiative than the commonly quoted 161 million estimate including 37 million blind, which excludes uncorrected refractive error.

Even for countries for which data on the contribution of uncorrected refractive error to visual impairment were available in this assessment, clear description of the method of attributing visual impairment to uncorrected refractive error and the types of refractive error leading to visual impairment was often missing. This could have led to under- or over-estimation of the contribution of uncorrected refractive error to the visual impairment burden. For example, under-estimation could have occurred in studies that did not include proper refraction, and over-estimation in studies that included substantial index myopia (induced by development of nuclear cataract) as a refractive error cause of visual impairment – the correct cause in such cases would be cataract. This situation points to the need to develop a standardised system for assessing the contribution of uncorrected refractive error to visual impairment in population-based studies, including distinguishing index myopia from natural refractive error and documentation of refractive error-related amblyopia [5].

It has to be kept in mind that the visual impairment definitions are currently based on distance visual acuity. A subset of the persons who have poor distance vision due to uncorrected refractive error, and qualify as visually impaired, may have good near vision. The difference between the impact on quality of life due to visual impairment caused by uncorrected refractive error that is associated with good near vision, and that due to visual impairment causing poor vision both at distance and near, needs to be better understood. On the other hand, it should also be noted that uncorrected presbyopia, refractive error due to aging that causes difficulty in seeing at near which usually starts progressing around 40 years of age, also causes disability. However, at this stage, the data available are not adequate to enable presbyopia-related visual impairment to be included in the definitions of visual impairment. It would be useful for such data to become available over a period of time.

We recommend that all population-based assessments of blindness and less severe visual impairment be based on presenting visual acuity so that uncorrected refractive error as a cause is not missed. In addition, if standardised requirements for reporting of visual impairment and its causes from population-based surveys were developed and implemented through a combined effort of journal editors, this would facilitate more efficient utilisation of future data for systematic tracking of visual impairment around the world.

## Conclusion

Although data on the contribution of uncorrected refractive error to visual impairment worldwide are scanty, our indicative estimate based on the available data suggests that the total number of persons with visual impairment in the world, who have presenting visual acuity less than 6/18 in the better eye, is about 259 million. This includes 42 million blind persons with presenting visual acuity less than 3/60 in the better eye and 217 million with less severe visual impairment of presenting visual acuity less than 6/18 to 3/60 in the better eye. This estimate of visually impaired persons is 61% higher than the 161 million estimate recently made by WHO on the basis of best-corrected visual acuity, which excluded visual impairment caused by uncorrected refractive error. Our estimate of 98 million persons in the world with visual impairment due to uncorrected refractive error makes this the largest cause of visual impairment. Estimates of visual impairment that include uncorrected refractive error must be used to avoid misleading underestimates from becoming the basis of planning for visual impairment reduction globally. We therefore suggest that the indicative estimate of 259 million persons in the world with visual impairment, which includes 42 million blind with visual acuity less than 3/60 in the better eye, be used for further planning of the VISION 2020 initiative instead of the commonly quoted recent WHO estimate of 161 million, which includes 37 million blind.

## Competing interests

The authors declare that they have no competing interests.

## Authors' contributions

LD conceived the idea of this report, reviewed the literature, designed the analysis and wrote the initial draft of the manuscript. RD contributed to the idea, analysis and writing of the manuscript. Both authors approved the final version of the manuscript.

## Pre-publication history

The pre-publication history for this paper can be accessed here:



## Supplementary Material

Additional File 1 Literature search results. Short description: This file lists each of the 283 publications that were assessed along with the reason assigned to each for inclusion or exclusion from our analysis. Click here for file

## Figures and Tables

**Table 1:** Number of qualifying surveys available for Global Burden of Disease (GBD) sub-regions.

GBD sub-region*	**WHO member countries**	**Population† **(millions)	**Number of qualifying surveys**
**Africa region**			
AFRO D	Algeria, Angola, Benin, Burkina Faso, Cameroon, Cape Verde, Chad, Comoros, Djibouti, Equatorial Guinea, Gabon, Gambia, Ghana, Guinea, Guinea-Bissau, Liberia, Madagascar, Mali, Mauritania, Mauritius, Niger, Nigeria, Sao Tome and Principe, Senegal, Seychelles, Sierra Leone, Somalia, Sudan, Togo	354	0
AFRO E	Botswana, Burundi, Central African Republic, Congo, Côte d'Ivoire, Democratic Republic of The Congo, Eritrea, Ethiopia, Kenya, Lesotho, Malawi, Mozambique, Namibia, Rwanda, South Africa, Swaziland, Uganda, United Republic of Tanzania, Zambia, Zimbabwe	361	1
**Americas region**			
AMRO A	Canada, United States of America	322	0
AMRO B	Antigua and Barbuda, Argentina, Bahamas, Barbados, Belize, Brazil, Chile, Colombia, Costa Rica, Cuba, Dominica, Dominican Republic, El Salvador, Grenada, Guyana, Honduras, Jamaica, Mexico, Panama, Paraguay, Saint Kitts and Nevis, Saint Lucia, Saint Vincent and The Grenadines, Suriname, Trinidad and Tobago, Uruguay, Venezuela	456	0
AMRO D	Bolivia, Ecuador, Guatemala, Haiti, Nicaragua, Peru	74	0
**Eastern Mediterranean region**			
EMRO B	Bahrain, Cyprus, Iran (Islamic Republic of), Jordan, Kuwait, Lebanon, Libyan Arab Jamahiriya, Oman, Qatar, Saudi Arabia, Syrian Arab Republic, Tunisia, United Arab Emirates	143	1
EMRO D	Egypt, Iraq, Morocco, Yemen	144	0
**Europe region**			
EURO A	Andorra, Austria, Belgium, Croatia, Czech Republic, Denmark, Finland, France, Germany, Greece, Iceland, Ireland, Israel, Italy, Luxembourg, Malta, Monaco, Netherlands, Norway, Portugal, San Marino, Slovenia, Spain, Sweden, Switzerland, United Kingdom	415	0
EURO B1	Albania, Bosnia and Herzegovina, Bulgaria, Georgia, Poland, Romania, Slovakia, The Former Yugoslav Republic of Macedonia, Turkey, Yugoslavia	170	1
EURO B2	Armenia, Azerbaijan, Kyrgyzstan, Tajikistan, Turkmenistan, Uzbekistan	53	0
EURO C	Belarus, Estonia, Hungary, Kazakhstan, Latvia, Lithuania, Republic of Moldova, Russian Federation, Ukraine	240	0
**South-East Asia region**			
SEARO B	Brunei Darussalam, Indonesia, Malaysia, Philippines, Singapore, Sri Lanka, Thailand	405	1
SEARO D	Afghanistan, Bangladesh, Bhutan, India, Maldives, Nepal, Pakistan	1394	3
**Western Pacific region**			
WPRO A	Australia, Japan, New Zealand	151	1
WPRO B1	China, DPR Korea, Mongolia, Republic of Korea	1375	1
WPRO B2	Cambodia, Lao People's Democratic Republic, Myanmar, Vietnam	148	0
WPRO B3	Cook Islands, Fiji, Kiribati, Marshall Islands, Micronesia (Federated States of), Nauru, Niue, Palau, Papua New Guinea, Samoa, Solomon Islands, Tonga, Tuvalu, Vanuatu	8	0
**Total**		6213	9

*GBD sub-regions as per the GBD 2000 Project; the letter with each sub-region indicates mortality stratum: A is very low child mortality and low adult mortality, B is low child mortality and low adult mortality, C is low child mortality and high adult mortality, D is high child mortality and high adult mortality, E is high child mortality and very high adult mortality; EURO B and WPRO B sub-divided further to capture epidemiological differences; this classification aims at maximising the epidemiological homogeneity of sub-regions [7].

†Population based on United Nations estimates for 2002 [20], as used for the WHO base visual impairment estimates [1].

**Table 2:** Data on contribution of uncorrected refractive error to blindness and less severe visual impairment in qualifying surveys.

**GBD sub-region**	**Country**	**Year of publica-tion of study **[Ref]	**Number of participants in survey**	**Partici-pation rate of those sampled **(%)	**Age group **(years)	Blindness* rate (%)	**Percent of blindness due to un-corrected refractive error**	**Less severe visual impairment† rate **(%)	**Percent of less severe visual impairment due to uncorrected refractive error**	**Types of refractive error causing visual impairment‡**
AFRO E	Ethiopia	1997 [10]	7423	90	All ages	0.85	8.0	1.68	28.8	Unspecified refractive error, aphakia
EMRO B	Lebanon	1997 [11]	10148	90	All ages	0.60	12.6	3.90	50.0	Myopia, hyperopia, aphakia
EURO B1	Turkey	1996 [12]	7497	87	All ages	0.40	12.0	1.50	29.0	Unspecified refractive error, aphakia
SEARO B	Malaysia	2002 [13]	18027	69	All ages	0.29	10.4	2.44	56.1	Unspecified refractive error, aphakia
SEARO D	India	2001 [14] 2002 [15]	10293	87	All ages	1.34	14.9	8.59	45.0	Myopia, hyperopia, aphakia, pseudo-phakia, refractive error related amblyopia
SEARO D	Pakistan§	1998 [16]	1156	90	All ages	2.08	16.7	6.92	55.0	Unspecified refractive error
SEARO D	Pakistan	2005 [17]	1106	94	40 or more	1.90	14.3	8.05	29.2	Unspecified refractive error, aphakia
WPRO A	Australia	2001 [18]	4744	86	40 or more	0.16	0.0	1.56	37.6	Myopia, hyperopia
WPRO B1	China	1999 [19]	5342	83	50 or more	2.67	12.4	NA	NA	Unspecified refractive error

*Blindness defined as presenting visual acuity less than 3/60; these studies, with two exceptions, did not include visual field loss criteria for blindness.

†Less severe visual impairment defined as presenting visual acuity less than 6/18 to 3/60.

‡Visual impairment defined as presenting visual acuity less than 6/18, which includes both blindness and less severe visual impairment.

§This study was on Afghan refugees in Pakistan; this would reflect more the visual impairment in Afghanistan, which is also in the SEARO D sub-region according to the GBD classification.

NA stands for not available.

**Table 3:** Estimation of blindness and visual impairment including that caused by uncorrected refractive error in different GBD sub-regions.

**GBD sub-region**	**Population **(millions)	Number blind with ICD definition* (millions)	Number having low vision with ICD definition* (millions)	Total number having visual impairment with ICD definition* (millions)	**Proportion of blindness estimated due to uncorrected refractive error**	**Proportion of less severe visual impairment estimated due to uncorrected refractive error**	**Number blind including un-corrected refractive error† **(millions)	**Number having less severe visual impairment including uncorrected refractive error† **(millions)	**Total number having visual impairment including uncorrected refractive error† **(millions)	**Increase in number with visual impairment due to uncorrected refractive error **(millions)
AFRO D‡	354	3.65	10.72	14.36	0.08	0.29	3.96	15.09	19.05	4.69
AFRO E	361	3.64	10.57	14.22	0.08	0.29	3.96	14.89	18.85	4.64
AMR A§	322	0.69	4.03	4.72	0	0.38	0.69	6.50	7.19	2.47
AMR B¶	456	1.39	7.60	8.99	0.10	0.45	1.55	13.82	15.36	6.37
AMR D#	74	0.33	1.49	1.82	0.15	0.45	0.39	2.71	3.10	1.28
EMRO B**	143	1.08	3.58	4.66	0.13	0.45	1.24	6.51	7.75	3.09
EMRO D#	144	1.41	4.12	5.52	0.15	0.45	1.65	7.48	9.14	3.62
EURO A§	415	0.94	5.44	6.37	0	0.38	0.94	8.77	9.70	3.33
EURO B1	170	0.62	2.55	3.16	0.12	0.29	0.70	3.59	4.29	1.12
EURO B2††	53	0.14	0.59	0.73	0.12	0.29	0.16	0.83	0.99	0.26
EURO C††	240	1.04	4.22	5.25	0.12	0.29	1.18	5.94	7.12	1.86
SEARO B‡‡	405	4.21	9.67	13.88	0.10	0.45	4.68	17.58	22.26	8.38
SEARO D§§	1394	8.34	28.44	36.78	0.15	0.45	9.82	51.71	61.52	24.74
WPRO A§	151	0.39	1.88	2.28	0	0.38	0.39	3.04	3.43	1.15
WPRO B1¶¶	1375	7.73	26.40	34.13	0.16	0.50	9.20	52.79	62.00	27.87
WPRO B2¶¶	148	1.23	2.90	4.13	0.16	0.50	1.46	5.80	7.26	3.13
WPRO B3¶¶	8	0.03	0.09	0.12	0.12	0.40	0.03	0.15	0.18	0.06
Total	6213	36.86	124.27	161.12	0.12	0.43	42.01	217.19	259.19	98.07

Some totals may not match exactly due to rounding off.

*These numbers with blindness (best-corrected visual acuity less than 3/60), low vision (best-corrected visual acuity less than 6/18 to 3/60) and visual impairment (best-corrected visual acuity less than 6/18) are from the WHO estimate using the ICD classification [1].

†Number of blind including uncorrected refractive error = Number of blind with ICD definition/(1 - Proportion of blindness estimated due to uncorrected refractive error); Number having less severe visual impairment including uncorrected refractive error = Number having low vision with ICD definition/(1 - Proportion of less severe visual impairment estimated due to uncorrected refractive error); total number having visual impairment including uncorrected refractive error is sum of the previous two.

‡Data from a national survey in Gambia in AFRO D sub-region could not be used as refractive error as a cause of visual impairment was mixed with "other" causes [21]; although data regarding contribution of uncorrected refractive error to blindness and less severe visual impairment from a D mortality stratum sub-region (SEARO D) were available, data from AFRO E used for AFRO D because of higher likelihood of similarities between the two sub-regions from Africa.

§Data from WPRO A used for AMR A and EURO A because of similar mortality stratum and high development stage of these sub-regions; as data from the qualifying survey from Australia for WPRO A was on a population aged 40 years or more [18], the proportional contribution of uncorrected refractive error to blindness and less severe visual impairment in the 40+ age group was used for the whole population in the absence of data on younger age groups.

¶Data from SEARO B used for AMR B because of similar mortality stratum for these sub-regions.

#Data from SEARO D used for AMR D and EMRO D because of similar mortality stratum for these sub-regions.

**Data from the qualifying survey from Lebanon for EMRO B suggested that approximately 50% of those with less severe visual impairment had their vision improved to 6/18 or better with pinhole [11]; due to the approximate nature of this estimate, we used a conservative estimate of 45% contribution of uncorrected refractive error to less severe visual impairment for this sub-region.

††Data from EURO B1 used for EURO B2 and EURO C because of similarities among these sub-regions that are made up predominantly of the former Soviet Bloc countries.

‡‡Data from the qualifying survey from Malaysia for SEARO B suggested that 56.1% of the less severe visual impairment was due to uncorrected refractive error including aphakia [13]; as this was higher than in any other qualifying survey, in order to avoid use of extreme estimates we used a conservative estimate of 45% contribution of uncorrected refractive error to less severe visual impairment for this sub-region.

§§For SEARO D sub-region, of the two qualifying surveys on all age group populations (Table 2), the lower proportional contribution of uncorrected refractive error to blindness and less severe visual impairment was used for this region.

¶¶Data from the qualifying survey from China for WPRO B1 on a population aged 50 years or more suggested 12.4% contribution of uncorrected refractive error to blindness, and these data for less severe visual impairment were not available [19]; as data from India suggest that the proportional contribution of uncorrected refractive error to blindness is relatively higher when younger age groups are included since blindness due to large uncorrected natural refractive errors sets in at an earlier age [14, 22], and because populations of Chinese origin have the highest rates of myopia [23-25], we estimated 16% and 50% contributions of uncorrected refractive error to blindness and less severe visual impairment, respectively, in the population of all age groups for WPRO B1; data from WPRO B1 used for WPRO B2 because of similar populations in these two sub-regions; relatively lower contribution of uncorrected refractive error to visual impairment assumed for WPRO B3 than in WPRO B1 or B2 because of the predominantly native populations in WPRO B3 that may possibly have a relatively lower burden of refractive error.
