# Supplementary material for: What is the global burden of visual impairment?
Source: BMC Med. 2006 Mar 16;4:6. doi: 10.1186/1741-7015-4-6 (PMC1435918; doi:10.1186/1741-7015-4-6)
Supplement: Additional File 1 — Literature search results. Short description: This file lists each of the 283 publications that were assessed along with the reason assigned to each for inclusion or exclusion from our analysis. [file 1741-7015-4-6-S1.pdf]

What is the global burden of visual impairment?

Additional file 1

Literature Search Results

PubMed and Cross References
Last PubMed search on 7 December 2005

Terms used for PubMed search:
blindness AND population-based survey
blindness AND population-based study
visual impairment AND population-based survey
visual impairment AND population-based study
low vision AND population-based survey
low vision AND population-based study
Limit:
Published 1996 onwards

List of publications located through PubMed and search of cross-references follows. Reason for inclusion or exclusion of each publication for utilising data for our analysis mentioned in bold below each publication.

1. Laitinen A, Koskinen S, Harkanen T, Reunanen A, Laatikainen L, Aromaa A. A nationwide population-based survey on visual acuity, near vision, and self-reported visual function in the adult population in Finland. Ophthalmology. 2005 Dec;112(12):2227-37. PMID: 16325714
   Excluded because: WHO categories of visual impairment not available

2. Thiagarajan M, Evans JR, Smeeth L, Wormald RP, Fletcher AE. Cause-specific visual impairment and mortality: results from a population-based study of older people in the United Kingdom. Arch Ophthalmol. 2005 Oct;123(10):1397-403. PMID: 16219731
   Excluded because: 75 years or older participants

3. Xu L, Li J, Cui T, Hu A, Zheng Y, Li Y, Sun B, Ma B, Jonas JB. Visual acuity in northern China in an urban and rural population: the Beijing Eye Study. Br J Ophthalmol. 2005 Sep;89(9):1089-93. PMID: 16113354
   Excluded because: Presenting visual acuity not available; uncorrected and best-corrected visual acuity reported

4. Robaei D, Rose K, Ojaimi E, Kifley A, Huynh S, Mitchell P. Visual acuity and the causes of visual loss in a population-based sample of 6-year-old Australian children. Ophthalmology. 2005 Jul;112(7):1275-82. PMID: 15921756
   Excluded because: children only

5. Taylor HR, Keeffe JE, Vu HT, Wang JJ, Rochtchina E, Pezzullo ML, Mitchell P.

Vision loss in Australia. Med J Aust. 2005 Jun 6;182(11):565-8. PMID: 15938683
Excluded because: WHO categories of visual impairment not available

6. Shaikh SP, Aziz TM. Pattern of eye diseases in children of 5-15 years at Bazzertaline Area (South Karachi) Pakistan. J Coll Physicians Surg Pak. 2005 May;15(5):291-4. PMID: 15907241
Excluded because: children only

7. He M, Xu J, Yin Q, Ellwein LB. Need and challenges of refractive correction in urban Chinese school children. Optom Vis Sci. 2005 Apr;82(4):229-34. PMID: 15829844
Excluded because: children only

8. Goh PP, Abqariyah Y, Pokharel GP, Ellwein LB. Refractive error and visual impairment in school-age children in Gombak District, Malaysia. Ophthalmology. 2005 Apr;112(4):678-85. PMID: 15808262
Excluded because: children only

9. Tsai CY, Woung LC, Chou P, Yang CS, Sheu MM, Wu JR, Chuang TL, Tung TH. The current status of visual disability in the elderly population of Taiwan. Jpn J Ophthalmol. 2005 Mar-Apr;49(2):166-72. PMID: 15838737
Excluded because: 65 years or older participants

10. Murthy GV, Gupta SK, Bachani D, Jose R, John N. Current estimates of blindness in India. Br J Ophthalmol. 2005 Mar;89(3):257-60. PMID: 15722298
Excluded because: discrepancy in contribution of refractive error to visual impairment as shown in two tables for which no explanation provided – Table 1 shows prevalence of presenting visual acuity less than 6/60 as 8.5% and prevalence of best-corrected visual acuity less than 6/60 as 4.3% (49% improvement from presenting acuity), but Table 3 mentions 19.6% of presenting vision less than 6/60 was due to refractive error and 1.5% of best-corrected vision less than 6/60 was due to refractive error(!); also, this study was on persons 50 years of age or older whereas other population-based studies on all age groups available from this country (India)

11. Ahmad K, Khan MD, Qureshi MB, Munami S, Shah RA, Rasheed H, Jamali B, Baluch A, Khan MA. Prevalence and causes of blindness and low vision in a rural setting in Pakistan. Ophthalmic Epidemiol. 2005 Feb;12(1):19-23. PMID: 15848917
INCLUDED – met inclusion criteria

12. Rudanko SL, Laatikainen L. Visual impairment in children born at full term from 1972 through 1989 in Finland. Ophthalmology. 2004 Dec;111(12):2307-12. PMID: 15582091
Excluded because: children only

13. Saw SM, Foster PJ, Gazzard G, Friedman D, Hee J, Seah S. Undercorrected refractive error in Singaporean Chinese adults: the Tanjong Pagar survey. Ophthalmology. 2004 Dec;111(12):2168-74. PMID: 15582070
Excluded because: data on causes of visual impairment from this survey presented in another paper

14. Adeoti CO. Prevalence and causes of blindness in a tropical African population. West Afr J Med. 2004 Jul-Sep;23(3):249-52. PMID: 15587840
Excluded because: number sampled not mentioned, making it difficult to determine the participation rate and generalisability of those who participated in this survey

15. Saw SM, Foster PJ, Gazzard G, Seah S. Causes of blindness, low vision, and questionnaire-assessed poor visual function in Singaporean Chinese adults: The Tanjong Pagar Survey. Ophthalmology. 2004 Jun;111(6):1161-8. PMID: 15177966
Excluded because: contribution of uncorrected refractive error to visual impairment not clear; prevalence of habitual bilateral low vision (presenting visual acuity less than 6/18 to 3/60) mentioned as 5.3% and prevalence of best-corrected visual acuity less than 6/18 to 3/60 mentioned as 1.1% (79% improvement from habitual acuity), but text mentions that 66% of subjects with bilateral low vision improved with best correction

16. Varma R, Ying-Lai M, Klein R, Azen SP; Los Angeles Latino Eye Study Group. Prevalence and risk indicators of visual impairment and blindness in Latinos: the Los Angeles Latino Eye Study. Ophthalmology. 2004 Jun;111(6):1132-40. PMID: 15177963
Excluded because: WHO categories of visual impairment not available

17. Fotouhi A, Hashemi H, Mohammad K, Jalali KH; Tehran Eye Study. The prevalence and causes of visual impairment in Tehran: the Tehran Eye Study. Br J Ophthalmol. 2004 Jun;88(6):740-5. PMID: 15148203
Excluded because: contribution of uncorrected refractive error not available separately for blindness and less severe visual impairment

18. Fouad D, Mousa A, Courtright P. Sociodemographic characteristics associated with blindness in a Nile Delta governorate of Egypt. Br J Ophthalmol. 2004 May;88(5):614-8. PMID: 15090410
Excluded because: contribution of uncorrected refractive error to visual impairment not distinct

19. Congdon N, O'Colmain B, Klaver CC, Klein R, Munoz B, Friedman DS, Kempen J, Taylor HR, Mitchell P; Eye Diseases Prevalence Research Group. Causes and prevalence of visual impairment among adults in the United States. Arch Ophthalmol. 2004 Apr;122(4):477-85. PMID: 15078664
Excluded because: best-corrected visual impairment definitions

20. Pascolini D, Mariotti SP, Pokharel GP, Pararajasegaram R, Etya'ale D, Negrel AD, Resnikoff S. 2002 global update of available data on visual impairment: a compilation of population-based prevalence studies. Ophthalmic Epidemiol. 2004 Apr;11(2):67-115. PMID: 15255026
Excluded because: this was a review and did not report primary data

21. Leske MC, Wu SY, Hyman L, Nemesure B, Hennis A, Schachat AP; Barbados Eye Studies Group. Four-year incidence of visual impairment: Barbados Incidence Study of Eye Diseases. Ophthalmology. 2004 Jan;111(1):118-24. PMID: 14711723
Excluded because: best-corrected visual impairment definitions

22. Hsu WM, Cheng CY, Liu JH, Tsai SY, Chou P. Prevalence and causes of visual impairment in an elderly Chinese population in Taiwan: the Shihpai Eye Study. Ophthalmology. 2004 Jan;111(1):62-9. PMID: 14711715
Excluded because: 65 years or older participants

23. Buch H, Vinding T, La Cour M, Appleyard M, Jensen GB, Nielsen NV. Prevalence and causes of visual impairment and blindness among 9980 Scandinavian adults: the Copenhagen City Eye Study. Ophthalmology. 2004 Jan;111(1):53-61. PMID: 14711714
Excluded because: best-corrected visual impairment definitions

24. Dunzhu S, Wang FS, Courtright P, Liu L, Tenzing C, Noertjojo K, Wilkie A, Santangelo M, Bassett KL. Blindness and eye diseases in Tibet: findings from a randomised, population based survey. Br J Ophthalmol. 2003 Dec;87(12):1443-8. PMID: 14660448
Excluded because: contribution of uncorrected refractive error to WHO categories of visual impairment not available

25. Nirmalan PK, Vijayalakshmi P, Sheeladevi S, Kothari MB, Sundaresan K, Rahmathullah L. The Kariapatti pediatric eye evaluation project: baseline ophthalmic data of children aged 15 years or younger in Southern India. Am J Ophthalmol. 2003 Oct;136(4):703-9. PMID: 14516811
Excluded because: children only

26. Stang A, Jockel KH. Visual disturbances in a population-based survey of 6962 subjects: the German National Health Examination Survey 1998. Eur J Public Health. 2003 Sep;13(3):202-9. PMID: 14533721
Excluded because: no visual acuity measurements reported

27. Courtright P, Hoechsmann A, Metcalfe N, Chirambo M, Noertjojo K, Barrows J, Katz J; Chikwawa Survey Team. Changes in blindness prevalence over 16 years in Malawi: reduced prevalence but increased numbers of blind. Br J Ophthalmol. 2003 Sep;87(9):1079-82. Erratum in: Br J Ophthalmol. 2003 Nov;87(11):1432.

Hoeshcmann, A [corrected to Hoechsmann, A]. PMID: 12928269
Excluded because: WHO categories of visual impairment not available

28. Saw SM, Husain R, Gazzard GM, Koh D, Widjaja D, Tan DT. Causes of low vision and blindness in rural Indonesia. Br J Ophthalmol. 2003 Sep;87(9):1075-8. PMID: 12928268
Excluded because: participating sample less than 1000

29. Thulasiraj RD, Nirmalan PK, Ramakrishnan R, Krishnadas R, Manimekalai TK, Baburajan NP, Katz J, Tielsch JM, Robin AL. Blindness and vision impairment in a rural south Indian population: the Aravind Comprehensive Eye Survey. Ophthalmology. 2003 Aug;110(8):1491-8. PMID: 12917162
Excluded because: contribution of uncorrected refractive error to visual impairment categories not clear – Table 4 mentions prevalence of presenting visual acuity less than 3/60 in persons 40 years or older as 4.5% and best-corrected visual acuity less than 3/60 as 1.0% (78% improvement from presenting acuity) – all of this 78% cannot be attribute to uncorrected refractive error as it seems implausibly high, and no explanation is provided in the paper about what else could have been responsible for this or the exact contribution of uncorrected refractive error to blindness / visual impairment

30. Chen JH, Xu L, Hu AL, Sun BC, Li JJ, Ma K, Xia CR, Cui TT, Zheng YY, Li YB, Zhang RX, Yang H, Sun XY, Zou Y, Wang Y, Ma BR. [Prevalence of low vision and blindness in defined populations in rural and urban areas in Beijing] Zhonghua Yi Xue Za Zhi. 2003 Aug 25;83(16):1413-8. Chinese. PMID: 14521745
Excluded because: results reported with best-corrected visual acuity

31. Dineen BP, Bourne RR, Ali SM, Huq DM, Johnson GJ. Prevalence and causes of blindness and visual impairment in Bangladeshi adults: results of the National Blindness and Low Vision Survey of Bangladesh. Br J Ophthalmol. 2003 Jul;87(7):820-8. PMID: 12812875
Excluded because: contribution of uncorrected refractive error to visual impairment not shown for the WHO categories after adjusting for the presence of cataract grade 2A or higher (the method used in this paper for attributing vision loss to uncorrected refractive error)

32. Melese M, Alemayehu W, Bayu S, Girma T, Hailesellasie T, Khandekar R, Worku A, Courtright P. Low vision and blindness in adults in Gurage Zone, central Ethiopia. Br J Ophthalmol. 2003 Jun;87(6):677-80. PMID: 12770959
Excluded because: the total number of visually impaired persons mentioned in the text and the numbers shown for the causes of visual impairment in Table 3 do not match; the latter may not be for causes of visual impairment in persons and therefore could not be used

33. Cedrone C, Culasso F, Cesareo M, Nucci C, Palma S, Mancino R, Cerulli L. Incidence of blindness and low vision in a sample population: the Priverno Eye Study, Italy. Ophthalmology. 2003 Mar;110(3):584-8. PMID: 12623826
Excluded because: best-corrected visual impairment definitions

34. Dandona R, Dandona L. Childhood blindness in India: a population based perspective. Br J Ophthalmol. 2003 Mar;87(3):263-5. PMID: 12598433
Excluded because: children only

35. Foran S, Mitchell P, Wang JJ. Five-year change in visual acuity and incidence of visual impairment: the Blue Mountains Eye Study. Ophthalmology. 2003 Jan;110(1):41-50. PMID: 12511344
Excluded because: WHO categories of visual impairment not available

36. Thulasiraj RD, Rahamathulla R, Saraswati A, Selvaraj S, Ellwein LB. The Sivaganga eye survey: I. Blindness and cataract surgery. Ophthalmic Epidemiol. 2002 Dec;9(5):299-312. PMID: 12528915
Excluded because: this study was on those 50 years of age or older, whereas other population-based studies on all age groups available from this country (India)

37. Dandona R, Dandona L, Srinivas M, Giridhar P, Nutheti R, Rao GN. Planning low vision services in India: a population-based perspective. Ophthalmology. 2002 Oct;109(10): 1871-8. PMID: 12359608
Excluded because: this paper used a low vision definition different from that used by WHO

38. Zainal M, Ismail SM, Ropilah AR, Elias H, Arumugam G, Alias D, Fathilah J, Lim TO, Ding LM, Goh PP. Prevalence of blindness and low vision in Malaysian population: results from the National Eye Survey 1996. Br J Ophthalmol. 2002 Sep;86(9):951-6. PMID: 12185113
INCLUDED – met inclusion criteria

39. Abdu L. Prevalence and causes of blindness and low vision in Dambatta local government area, Kano State, Nigeria. Niger J Med. 2002 Jul-Sep;11(3):108-12. PMID: 12221951
Excluded because: basis for attributing visual impairment to refractive error not clear

40. Foran S, Wang JJ, Mitchell P. Causes of incident visual impairment: the Blue Mountains Eye Study. Arch Ophthalmol. 2002 May;120(5):613-9. PMID: 12003611
Excluded because: WHO categories of visual impairment not available

41. Nirmalan PK, Thulasiraj RD, Maneksha V, Rahmathullah R, Ramakrishnan R, Padmavathi A, Munoz SR, Ellwein LB. A population based eye survey of older

adults in Tirunelveli district of south India: blindness, cataract surgery, and visual outcomes. Br J Ophthalmol. 2002 May;86(5):505-12. PMID: 11973242
Excluded because: this study was on those 50 years of age or older, whereas other population-based studies on all age groups available from this country (India)

42. Dandona R, Dandona L, Srinivas M, Giridhar P, Prasad MN, Vilas K, McCarty CA, Rao GN. Moderate visual impairment in India: the Andhra Pradesh Eye Disease Study. Br J Ophthalmol. 2002 Apr;86(4):373-7. PMID: 11914201
INCLUDED – met inclusion criteria

43. Rodriguez J, Sanchez R, Munoz B, West SK, Broman A, Snyder RW, Klein R, Quigley H. Causes of blindness and visual impairment in a population-based sample of U.S. Hispanics. Ophthalmology. 2002 Apr;109(4):737-43. PMID: 11927431
Excluded because: best-corrected visual impairment definitions

44. Haddadin A, Ereifej I, Zawaida F, Haddadin H. Causes of visual impairment and blindness among the middle-aged and elderly in northern Jordan. East Mediterr Health J. 2002 Mar-May;8(2-3):404-8. PMID: 15339130
Excluded because: hospital-based data

45. Munoz B, West SK, Rodriguez J, Sanchez R, Broman AT, Snyder R, Klein R. Blindness, visual impairment and the problem of uncorrected refractive error in a Mexican-American population: Proyecto VER. Invest Ophthalmol Vis Sci. 2002 Mar;43(3):608-14. PMID: 11867574
Excluded because: contribution of uncorrected refractive error to WHO categories of visual impairment not available

46. Michon JJ, Lau J, Chan WS, Ellwein LB. Prevalence of visual impairment, blindness, and cataract surgery in the Hong Kong elderly. Br J Ophthalmol. 2002 Feb;86(2):133-9. PMID: 11815334
Excluded because: 60 years or older participants

47. Dandona R, Dandona L. Review of findings of the Andhra Pradesh Eye Disease Study: policy implications for eye-care services. Indian J Ophthalmol. 2001 Dec;49(4):215-34. PMID: 12930114
Excluded because: primary data from this study in other papers included

48. Liu JH, Cheng CY, Chen SJ, Lee FL. Visual impairment in a Taiwanese population: prevalence, causes, and socioeconomic factors. Ophthalmic Epidemiol. 2001 Dec;8(5): 339-50. PMID: 11922386
Excluded because: best-corrected visual impairment definitions

49. Dandona L, Dandona R, John RK. Estimation of blindness in India from 2000 through 2020: implications for the blindness control policy. Natl Med J India.

2001 Nov-Dec;14 (6):327-34. PMID: 11804362
Excluded because: primary data from this study in other papers included

50. Klein R, Klein BE, Lee KE, Cruickshanks KJ, Chappell RJ. Changes in visual acuity in a population over a 10-year period : The Beaver Dam Eye Study. Ophthalmology. 2001 Oct;108(10):1757-66. PMID: 11581046
Excluded because: WHO categories of visual impairment not available

51. Hyman L, Wu SY, Connell AM, Schachat A, Nemesure B, Hennis A, Leske MC. Prevalence and causes of visual impairment in The Barbados Eye Study. Ophthalmology. 2001 Oct;108(10):1751-6. PMID: 11581045
Excluded because: best-corrected visual impairment definitions

52. Lewallen S, Courtright P. Blindness in Africa: present situation and future needs. Br J Ophthalmol. 2001 Aug;85(8):897-903. Review. PMID: 11466240
Excluded because: this was a review and did not report primary data

53. VanNewkirk MR, Weih L, McCarty CA, Taylor HR. Cause-specific prevalence of bilateral visual impairment in Victoria, Australia: the Visual Impairment Project. Ophthalmology. 2001 May;108(5):960-7. PMID: 11320028
INCLUDED – met eligibility criteria

54. Murthy GV, Gupta S, Ellwein LB, Munoz SR, Bachani D, Dada VK. A population-based eye survey of older adults in a rural district of Rajasthan: I. Central vision impairment, blindness, and cataract surgery. Ophthalmology. 2001 Apr;108(4):679-85. PMID: 11297483
Excluded because: this study was on those 50 years of age or older, whereas other population-based studies on all age groups available from this country (India)

55. Dandona L, Dandona R, Srinivas M, Giridhar P, Vilas K, Prasad MN, John RK, McCarty CA, Rao GN. Blindness in the Indian state of Andhra Pradesh. Invest Ophthalmol Vis Sci. 2001 Apr;42(5):908-16. PMID: 11274066
INCLUDED – met eligibility criteria

56. Abou-Gareeb I, Lewallen S, Bassett K, Courtright P. Gender and blindness: a meta-analysis of population-based prevalence surveys. Ophthalmic Epidemiol. 2001 Feb;8(1):39-56. PMID: 11262681
Excluded because: this was a review and did not report primary data

57. Faal H, Minassian DC, Dolin PJ, Mohamed AA, Ajewole J, Johnson GJ. Evaluation of a national eye care programme: re-survey after 10 years. Br J Ophthalmol. 2000 Sep;84(9):948-51. PMID: 10966942
Excluded because: refractive error as a cause of visual impairment shown mixed with the "others" category and not shown distinctly

58. Munoz B, West SK, Rubin GS, Schein OD, Quigley HA, Bressler SB, Bandeen-Roche K. Causes of blindness and visual impairment in a population of older Americans: The Salisbury Eye Evaluation Study. Arch Ophthalmol. 2000 Jun;118(6):819-25. PMID: 10865321
Excluded because: 65-84 years old participants

59. Foran S, Wang JJ, Rochtchina E, Mitchell P. Projected number of Australians with visual impairment in 2000 and 2030. Clin Experiment Ophthalmol. 2000 Jun;28(3):143-5. PMID: 10981782
Excluded because: primary data on visual impairment and its causes not reported in this paper

60. Cohen D, Sartral M, Nounou P, Hamar M, Drouard ME, El Alamy A, Bendeddouche K. [Evaluation of moderate and severe visual impairments in patients attending an ophthalmology clinic. A prospective study of 1,172 patients] J Fr Ophtalmol. 2000 May;23(5):437-43. French. PMID: 10844301
Excluded because: hospital-based study

61. Lam BL, Lee DJ, Gomez-Marin O. Prevalence of usual-corrected binocular distance visual acuity impairment in Hispanic and non-Hispanic adults. Ophthalmic Epidemiol. 2000 Mar;7(1):73-83. PMID: 10652174
Excluded because: WHO categories of visual impairment not available

62. van der Pols JC, Bates CJ, McGraw PV, Thompson JR, Reacher M, Prentice A, Finch S. Visual acuity measurements in a national sample of British elderly people. Br J Ophthalmol. 2000 Feb;84(2):165-70. PMID: 10655192
Excluded because: 65 years or older participants

63. Li S, Xu J, He M, Wu K, Munoz SR, Ellwein LB. A survey of blindness and cataract surgery in Doumen County, China. Ophthalmology. 1999 Aug;106(8):1602-8. PMID: 10442910
INCLUDED – met inclusion criteria

64. Bergman B, Bergstrom A, Sjostrand J. Longitudinal changes in visual acuity and visual ability in a cohort followed from the age of 70 to 88 years. Acta Ophthalmol Scand. 1999 Jun;77(3):286-92. PMID: 10406147
Excluded because: 70-88 years old participants

65. Dandona L, Dandona R, Naduvilath TJ, McCarty CA, Srinivas M, Mandal P, Nanda A, Rao GN. Burden of moderate visual impairment in an urban population in southern India. Ophthalmology. 1999 Mar;106(3):497-504. PMID: 10080205
Excluded because: later paper with both urban and rural data from this study included

66. Krumpaszky HG, Ludtke R, Mickler A, Klauss V, Selbmann HK. Blindness incidence in Germany. A population-based study from Wurttemberg-

Hohenzollern. Ophthalmologica. 1999;213(3):176-82. PMID: 10202291
Excluded because: WHO categories of visual impairment not available

67. Awan HR, Ihsan T. Prevalence of visual impairment and eye diseases in Afghan refugees in Pakistan. East Mediterr Health J. 1998;4(3):560-6.
INCLUDED – met inclusion criteria

68. Zhao J, Jia L, Sui R, Ellwein LB. Prevalence of blindness and cataract surgery in Shunyi County, China. Am J Ophthalmol. 1998 Oct;126(4):506-14. PMID: 9780095
Excluded because: contribution of uncorrected refractive error to visual impairment in persons not clear

69. Ayed S, Negrel AD, Nabli M, Kamel N, Jebri AM, Siddhom M. [Prevalence and causes of blindness in the Tunisian Republic. Results of a national survey conducted in 1993. Tunisian Team on the Evaluation of Blindness] Sante. 1998 Jul-Aug;8(4):275-82. French. PMID: 9794038
Excluded because: contribution of uncorrected refractive error to visual impairment not clear

70. Dandona L, Dandona R, Naduvilath TJ, McCarty CA, Nanda A, Srinivas M, Mandal P, Rao GN. Is current eye-care-policy focus almost exclusively on cataract adequate to deal with blindness in India? Lancet. 1998 May 2;351(9112):1312-6. PMID: 9643793
Excluded because: later paper with both urban and rural data from this study included

71. Reidy A, Minassian DC, Vafidis G, Joseph J, Farrow S, Wu J, Desai P, Connolly A. Prevalence of serious eye disease and visual impairment in a north London population: population based, cross sectional study. BMJ. 1998 May 30;316(7145):1643-6. PMID: 9603746
Excluded because: 65 years or older participants

72. Klaver CC, Wolfs RC, Vingerling JR, Hofman A, de Jong PT. Age-specific prevalence and causes of blindness and visual impairment in an older population: the Rotterdam Study. Arch Ophthalmol. 1998 May;116(5):653-8. PMID: 9596502
Excluded because: best-corrected visual impairment definitions

73. Dandona L, Williams JD, Williams BC, Rao GN. Population-based assessment of childhood blindness in southern India. Arch Ophthalmol. 1998 Apr;116(4):545-6. No abstract available. PMID: 9565065
Excluded because: children only

74. Zainal M, Masran L, Ropilah AR. Blindness and visual impairment amongst rural Malays in Kuala Selangor, Selangor. Med J Malaysia. 1998 Mar;53(1):46-50.

PMID: 10968137
Excluded because: participating sample size less than 1000

75. Mansour AM, Kassak K, Chaya M, Hourani T, Sibai A, Alameddine MN. National survey of blindness and low vision in Lebanon. Br J Ophthalmol. 1997 Oct;81(10):905-6. PMID: 9486035
INCLUDED – met inclusion criteria

76. Ezepue UF. Magnitude and causes of blindness and low vision in Anambra State of Nigeria (results of 1992 point prevalence survey). Public Health. 1997 Sep;111(5):305-9. PMID: 9308379
Excluded because: best-corrected visual impairment definitions

77. Schwartz EC, Huss R, Hopkins A, Dadjim B, Madjitoloum P, Henault C, Klauss V. Blindness and visual impairment in a region endemic for onchocerciasis in the Central African Republic. Br J Ophthalmol. 1997 Jun;81(6):443-7. PMID: 9274406
Excluded because: data from this atypical area could not be generalised to the GDB sub-region

78. Stocks NP, Hiller JE, Newland H. Visual acuity in an Australian aboriginal population. Aust N Z J Ophthalmol. 1997 May;25(2):125-31. PMID: 9267598
Excluded because: data atypical for this predominantly well-developed GDB sub-region

79. Jackson H, Foster A. Causes of blindness in northwest Cambodia. Ophthalmic Epidemiol. 1997 Mar;4(1):27-32. PMID: 9145413
Excluded because: hospital-based data

80. Zerihun N, Mabey D. Blindness and low vision in Jimma Zone, Ethopia: results of a population-based survey. Ophthalmic Epidemiol. 1997 Mar;4(1):19-26. PMID: 9145412
INCLUDED – met eligibility criteria

81. Taylor HR, Livingston PM, Stanislavsky YL, McCarty CA. Visual impairment in Australia: distance visual acuity, near vision, and visual field findings of the Melbourne Visual Impairment Project. Am J Ophthalmol. 1997 Mar;123(3):328-37. PMID: 9063242
Excluded because: later paper from this study included

82. Krumpaszky HG. [Temporal trends in the etiology of blindness] Klin Monatsbl Augenheilkd. 1997 Feb;210(2):aA9-16. Review. German. PMID: 9229589
Excluded because: contribution of uncorrected refractive error to visual impairment not clear

83. West SK, Munoz B, Rubin GS, Schein OD, Bandeen-Roche K, Zeger S, German S, Fried LP. Function and visual impairment in a population-based study of older adults. The SEE project. Salisbury Eye Evaluation. Invest Ophthalmol Vis Sci. 1997 Jan;38(1):72-82. PMID: 9008632
Excluded because: 65-84 years old participants

84. Van Newkirk MR. The Hong Kong vision study: a pilot assessment of visual impairment in adults. Trans Am Ophthalmol Soc. 1997;95:715-49. PMID: 9440191
Excluded because: participating sample size less than 1000

85. Vassileva P, Gieser SC, Vitale S, Cholakova T, Katz J, West S. Blindness and visual impairment in western Bulgaria. Ophthalmic Epidemiol. 1996 Dec;3(3):143-9. PMID: 8956318
Excluded because: contribution of uncorrected refractive error to visual impairment in persons not clear

86. Negrel AD, Minassian DC, Sayek F. Blindness and low vision in southeast Turkey. Ophthalmic Epidemiol. 1996 Dec;3(3):127-34. PMID: 8956316
INCLUDED – met inclusion criteria

87. Haussler M, Bartels H, Strassburg HM. Multihandicapped blind and partially sighted children in south Germany. II: Aetiology and pathogenesis. Dev Med Child Neurol. 1996 Dec;38(12):1076-90. PMID: 8973293
Excluded because: children only

88. Haussler M, Schafer WD, Neugebauer H. Multihandicapped blind and partially sighted children in south Germany. I: Prevalence, impairments and ophthalmological findings. Dev Med Child Neurol. 1996 Dec;38(12):1068-75. PMID: 8973292
Excluded because: children only

89. Rahmani B, Tielsch JM, Katz J, Gottsch J, Quigley H, Javitt J, Sommer A. The cause-specific prevalence of visual impairment in an urban population. The Baltimore Eye Survey. Ophthalmology. 1996 Nov;103(11):1721-6. PMID: 8942862
Excluded because: best-corrected visual impairment definitions

90. Adeoye A. Survey of blindness in rural communities of south-western Nigeria. Trop Med Int Health. 1996 Oct;1(5):672-6. PMID: 8911452
Excluded because: best-corrected visual impairment definitions

91. Casson R, Giles L, Newland HS. Prevalence of blindness and visual impairment in an elderly urban population. Aust N Z J Ophthalmol. 1996 Aug;24(3):239-43. PMID: 8913126
Excluded because: 70 years or older participants

92. Klein R, Klein BE, Lee KE. Changes in visual acuity in a population. The Beaver Dam Eye Study. Ophthalmology. 1996 Aug;103(8):1169-78. PMID: 8764783
Excluded because: WHO visual impairment categories not available

93. Newland HS, Hiller JE, Casson RJ, Obermeder S. Prevalence and causes of blindness in the South Australian population aged 50 and over. Ophthalmic Epidemiol. 1996 Jun;3 (2): 97-107. PMID: 8841061
Excluded because: data collected before 1991

94. Kortlang C, Koster JC, Coulibaly S, Dubbeldam RP. Prevalence of blindness and visual impairment in the region of Segou, Mali. A baseline survey for a primary eye care programme. Trop Med Int Health. 1996 Jun;1(3):314-9. PMID: 8673833
Excluded because: data collected before 1991

95. Wilson MR, Mansour M, Ross-Degnan D, Moukouri E, Fobi G, Alemayehu W, Martone JF, Casey R, Bazargan M. Prevalence and causes of low vision and blindness in the Extreme North Province of Cameroon, West Africa. Ophthalmic Epidemiol. 1996 Mar; 3(1):23-33. PMID: 8705870
Excluded because: "best" visual impairment definitions used but refractive error shown as cause for blindness or low vision combined and not separately; therefore, contribution of uncorrected refractive error to WHO categories of visual impairment not clear

96. Attebo K, Mitchell P, Smith W. Visual acuity and the causes of visual loss in Australia. The Blue Mountains Eye Study. Ophthalmology. 1996 Mar;103(3):357-64. PMID: 8600410
Excluded because: contribution of uncorrected refractive error to WHO categories of visual impairment not available

The following publications were excluded because these did not report data on population-based assessment of visual impairment and the proportional contribution of its various causes:

97. Smeeth L, Cook C, Chakravarthy U, Hubbard R, Fletcher AE. A case control study of age related macular degeneration and use of statins. Br J Ophthalmol. 2005 Sep; 89 (9): 1171-5. PMID: 16113375

98. van Splunder J, Stilma JS, Bernsen RM, Evenhuis HM. Prevalence of visual impairment in adults with intellectual disabilities in the Netherlands: cross-sectional study. Eye. 2005 Sep 9; [Epub ahead of print] PMID: 16151486

99. West SK, Munoz B, Rubin GS, Bandeen-Roche K, Broman AT, Turano KA. Compensatory strategy use identifies risk of incident disability for the visually impaired. Arch Ophthalmol. 2005 Sep;123(9):1242-7. PMID: 16157806

100. Donnelly UM, Stewart NM, Hollinger M. Prevalence and outcomes of childhood visual disorders. Ophthalmic Epidemiol. 2005 Aug;12(4):243-50. PMID: 16033745

101. McCarty DJ, Mukesh BN, Chikani V, Wang JJ, Mitchell P, Taylor HR, McCarty CA. Prevalence and associations of epiretinal membranes in the visual impairment project. Am J Ophthalmol. 2005 Aug;140(2):288-94. PMID: 16023066

102. Globe DR, Varma R, Torres M, Wu J, Klein R, Azen SP; Los Angeles Latino Eye Study Group. Self-reported comorbidities and visual function in a population-based study: the Los Angeles Latino Eye Study. Arch Ophthalmol. 2005 Jun;123(6):815-21. PMID: 15955983

103. Santangelo SL, Yen CH, Haddad S, Fagerness J, Huang C, Seddon JM. A discordant sib-pair linkage analysis of age-related macular degeneration. Ophthalmic Genet. 2005 Jun;26(2):61-7. PMID: 16020308

104. Rull JA, Aguilar-Salinas CA, Rojas R, Rios-Torres JM, Gomez-Perez FJ, Olaiz G. Epidemiology of type 2 diabetes in Mexico. Arch Med Res. 2005 May-Jun;36(3):188-96. Review. PMID: 15925009

105. Evans JR, Fletcher AE, Wormald RP. 28,000 Cases of age related macular degeneration causing visual loss in people aged 75 years and above in the United Kingdom may be attributable to smoking. Br J Ophthalmol. 2005 May;89(5):550-3. PMID: 15834082

106. Buch H, Nielsen NV, Vinding T, Jensen GB, Prause JU, la Cour M. 14-year incidence, progression, and visual morbidity of age-related maculopathy: the Copenhagen City Eye Study. Ophthalmology. 2005 May;112(5):787-98. PMID: 15878058

107. Haines L, Fielder AR, Baker H, Wilkinson AR. UK population based study of severe retinopathy of prematurity: screening, treatment, and outcome. Arch Dis Child Fetal Neonatal Ed. 2005 May;90(3):F240-4. PMID: 15846016

108. Thomas R, Nirmalan PK, Krishnaiah S. Pseudoexfoliation in southern India: the Andhra Pradesh Eye Disease Study. Invest Ophthalmol Vis Sci. 2005 Apr;46(4):1170-6. PMID: 15790875

109. Centers for Disease Control and Prevention (CDC). Diabetes-related preventive-care practices--Guam, 2001-2003. MMWR Morb Mortal Wkly Rep. 2005 Apr 8;54(13):333- 5. PMID: 15815564

110. Coleman AL, Yu F, Rowe S. Visual field testing in glaucoma Medicare beneficiaries before surgery. Ophthalmology. 2005 Mar;112(3):401-6. PMID: 15745765

111. Frick KD, Foster A, Bah M, Faal H. Analysis of costs and benefits of the Gambian Eye Care Program. Arch Ophthalmol. 2005 Feb;123(2):239-43. PMID: 15710822

112. Duggal P, Klein AP, Lee KE, Iyengar SK, Klein R, Bailey-Wilson JE, Klein BE. A genetic contribution to intraocular pressure: the beaver dam eye study. Invest Ophthalmol Vis Sci. 2005 Feb;46(2):555-60. PMID: 15671282

113. Baker RS, Bazargan M, Bazargan-Hejazi S, Calderon JL. Access to vision care in an urban low-income multiethnic population. Ophthalmic Epidemiol. 2005 Feb;12(1):1-12. PMID: 15848915

114. Lee DJ, Gomez-Marin O, Lam BL, Zheng DD, Caban A. Visual impairment and morbidity in community-residing adults: the national health interview survey 1986-1996. Ophthalmic Epidemiol. 2005 Feb;12(1):13-7. PMID: 15848916

115. Bassett KL, Noertjojo K, Liu L, Wang FS, Tenzing C, Wilkie A, Santangelo M, Courtright P. Cataract surgical coverage and outcome in the Tibet Autonomous Region of China. Br J Ophthalmol. 2005 Jan;89(1):5-9. PMID: 15615736

116. Vijayakumar V, John RK, Datta D, Thulasiraj RD, Nirmalan PK. Quality of life after community-based rehabilitation for blind persons in a rural population of South India. Indian J Ophthalmol. 2004 Dec;52(4):331-5. PMID: 15693330

117. Bylsma GW, Le A, Mukesh BN, Taylor HR, McCarty CA. Utilization of eye care services by Victorians likely to benefit from eye care. Clin Experiment Ophthalmol. 2004 Dec;32(6):573-7. PMID: 15575826

118. Rahman MM, Rahman N, Foster PJ, Haque Z, Zaman AU, Dineen B, Johnson GJ. The prevalence of glaucoma in Bangladesh: a population based survey in Dhaka division. Br J Ophthalmol. 2004 Dec;88(12):1493-7. PMID: 15548796

119. Evenhuis H, van Splunder J, Vink M, Weerdenburg C, van Zanten B, Stilma J. Obstacles in large-scale epidemiological assessment of sensory impairments in a Dutch population with intellectual disabilities. J Intellect Disabil Res. 2004 Nov;48(Pt 8):708-18. PMID: 15494060

120. Bohm B, Smedler AC, Forssberg H. Impulse control, working memory and other executive functions in preterm children when starting school. Acta Paediatr. 2004 Oct;93(10):1363-71. PMID: 15499959

121. Nutheti R, Shamanna BR, Krishnaiah S, Gothwal VK, Thomas R, Rao GN. Perceived visual ability for functional vision performance among persons with low vision in the Indian state of Andhra Pradesh. Invest Ophthalmol Vis Sci. 2004 Oct;45(10):3458-65. PMID: 15452050

122. Nirmalan PK, John RK, Gothwal VK, Baskaran S, Vijayalakshmi P, Rahmathullah L; Kariapatti Pediatric Eye Evaluation Project. The impact of visual impairment on functional vision of children in rural South India: the Kariapatti Pediatric Eye Evaluation Project. Invest Ophthalmol Vis Sci. 2004 Oct;45(10):3442-5. PMID: 15452047

123. Nirmalan PK, Katz J, Tielsch JM, Robin AL, Thulasiraj RD, Krishnadas R, Ramakrishnan R; Aravind Comprehensive Eye Survey. Ocular trauma in a rural south Indian population: the Aravind Comprehensive Eye Survey. Ophthalmology. 2004 Sep; 111(9):1778-81. PMID: 15350336

124. Chua B, Mitchell P. Consequences of amblyopia on education, occupation, and long term vision loss. Br J Ophthalmol. 2004 Sep;88(9):1119-21. PMID: 15317699

125. Varma R, Torres M; Los Angeles Latino Eye Study Group. Prevalence of lens opacities in Latinos: the Los Angeles Latino Eye Study. Ophthalmology. 2004 Aug;111(8):1449-56. PMID: 15288970

126. Mukesh BN, Dimitrov PN, Leikin S, Wang JJ, Mitchell P, McCarty CA, Taylor HR. Five-year incidence of age-related maculopathy: the Visual Impairment Project. Ophthalmology. 2004 Jun;111(6):1176-82. PMID: 15177968

127. Bourne RR, Dineen BP, Ali SM, Noorul Huq DM, Johnson GJ. Prevalence of refractive error in Bangladeshi adults: results of the National Blindness and Low Vision Survey of Bangladesh. Ophthalmology. 2004 Jun;111(6):1150-60. PMID: 15177965

128. Globe DR, Wu J, Azen SP, Varma R; Los Angeles Latino Eye Study Group. The impact of visual impairment on self-reported visual functioning in Latinos: The Los Angeles Latino Eye Study. Ophthalmology. 2004 Jun;111(6):1141-9. PMID: 15177964

129. Varma R, Paz SH, Azen SP, Klein R, Globe D, Torres M, Shufelt C, Preston-Martin S; Los Angeles Latino Eye Study Group. The Los Angeles Latino Eye Study: design, methods, and baseline data. Ophthalmology. 2004 Jun;111(6):1121-31. PMID: 15177962

130. Balo KP, Anika A, Banla M, Agla K, Djagnikpo PA, Koffi Gue KB. [The distribution of cup disc ratios in a general population of Southern Togo aged 40 years and over] J Fr Ophtalmol. 2004 Mar;27(3):250-5. French. PMID: 15039626

131. Evans JR, Fletcher AE, Wormald RP. Age-related macular degeneration causing visual impairment in people 75 years or older in Britain: an add-on study to the Medical research Council Trial of Assessment and Management of Older People in the Community. Ophthalmology. 2004 Mar;111(3):513-7. PMID: 15019328

132. Melese M, Alemayehu W, Friedlander E, Courtright P. Indirect costs associated with accessing eye care services as a barrier to service use in Ethiopia. Trop Med Int Health. 2004 Mar;9(3):426-31. PMID: 14996373

133. Vautour LM, Melton LJ 3rd, Clarke BL, Achenbach SJ, Oberg AL, McCarthy JT. Long-term fracture risk following renal transplantation: a population-based study. Osteoporos Int. 2004 Feb;15(2):160-7. Epub 2003 Dec 9. PMID: 14666400

134. Kemper AR, Bruckman D, Freed GL. Prevalence and distribution of corrective lenses among school-age children. Optom Vis Sci. 2004 Jan;81(1):7-10. PMID: 14747754

135. Gulliford MC, Mahabir D, Rocke B. Diabetes-related inequalities in health status and financial barriers to health care access in a population-based study. Diabet Med. 2004 Jan;21(1):45-51. PMID: 14706053

136. Lee DJ, Gomez-Marin O, Lam BL, Zheng DD. Visual impairment and unintentional injury mortality: the National Health Interview Survey 1986-1994. Am J Ophthalmol. 2003 Dec;136(6):1152-4. PMID: 14644228

137. Thomas R, Parikh R, George R, Kumar RS, Muliyil J. Five-year risk of progression of ocular hypertension to primary open angle glaucoma. A population-based study. Indian J Ophthalmol. 2003 Dec;51(4):329-33. PMID: 14750621

138. Nicolas CM, Robman LD, Tikellis G, Dimitrov PN, Dowrick A, Guymer RH, McCarty CA. Iris colour, ethnic origin and progression of age-related macular degeneration. Clin Experiment Ophthalmol. 2003 Dec;31(6):465-9. PMID: 14641151

139. Steffenburg S, Steffenburg U, Gillberg C. Autism spectrum disorders in children with active epilepsy and learning disability: comorbidity, pre- and perinatal background, and seizure characteristics. Dev Med Child Neurol. 2003 Nov;45(11):724-30. PMID: 14580127

140. West CG, Gildengorin G, Haegerstrom-Portnoy G, Lott LA, Schneck ME, Brabyn JA. Vision and driving self-restriction in older adults. J Am Geriatr Soc. 2003 Oct;51 (10): 1348-55. PMID: 14511153

141. Thomas R, Parikh R, Muliyil J, Kumar RS. Five-year risk of progression of primary angle closure to primary angle closure glaucoma: a population-based study. Acta Ophthalmol Scand. 2003 Oct;81(5):480-5. PMID: 14510795

142. Brown JB, Pedula KL, Summers KH. Diabetic retinopathy: contemporary prevalence in a well-controlled population. Diabetes Care. 2003 Sep;26(9):2637-42. PMID: 12941732

143. Ramakrishnan R, Nirmalan PK, Krishnadas R, Thulasiraj RD, Tielsch JM, Katz J, Friedman DS, Robin AL. Glaucoma in a rural population of southern India: the Aravind comprehensive eye survey. Ophthalmology. 2003 Aug;110(8):1484-90. Erratum in: Ophthalmology. 2004 Feb;111(2):331. PMID: 12917161

144. Paz SH, Globe DR, Wu J, Azen SP, Varma R; Los Angeles Latino Eye Study. Relationship between self-reported depression and self-reported visual function in Latinos. Arch Ophthalmol. 2003 Jul;121(7):1021-7. PMID: 12860807

145. Hashemi H, Fotouhi A, Mohammad K. The Tehran Eye Study: research design and eye examination protocol. BMC Ophthalmol. 2003 Jul 15;3:8. PMID: 12859794

146. Bourne RR, Dineen BP, Ali SM, Huq DM, Johnson GJ. Outcomes of cataract surgery in Bangladesh: results from a population based nationwide survey. Br J Ophthalmol. 2003 Jul;87(7):813-9. PMID: 12812874

147. Tsai SY, Hsu WM, Cheng CY, Liu JH, Chou P. Epidemiologic study of age-related cataracts among an elderly Chinese population in Shih-Pai, Taiwan. Ophthalmology. 2003 Jun;110(6):1089-95. PMID: 12799231

148. Krishnadas R, Nirmalan PK, Ramakrishnan R, Thulasiraj RD, Katz J, Tielsch JM, Friedman DS, Robin AL. Pseudoexfoliation in a rural population of southern India: the Aravind Comprehensive Eye Survey. Am J Ophthalmol. 2003 Jun;135(6):830-7. PMID: 12788123

149. Rochtchina E, Mukesh BN, Wang JJ, McCarty CA, Taylor HR, Mitchell P. Projected prevalence of age-related cataract and cataract surgery in Australia for the years 2001 and 2021: pooled data from two population-based surveys. Clin Experiment Ophthalmol. 2003 Jun;31(3):233-6. PMID: 12786774

150. Edelsten C, Reddy MA, Stanford MR, Graham EM. Visual loss associated with pediatric uveitis in english primary and referral centers. Am J Ophthalmol. 2003 May;135(5):676-80. PMID: 12719076

151. Bhatti TR, Dott M, Yoon PW, Moore CA, Gambrell D, Rasmussen SA. Descriptive epidemiology of infantile cataracts in metropolitan Atlanta, GA, 1968-1998. Arch Pediatr Adolesc Med. 2003 Apr;157(4):341-7. PMID: 12695229

152. Holmes JM, Leske DA, Burke JP, Hodge DO. Birth prevalence of visually significant infantile cataract in a defined U.S. population. Ophthalmic Epidemiol. 2003 Apr;10 (2): 67-74. PMID: 12660855

153. Globe D, Varma R, Azen SP, Paz S, Yu E, Preston-Martin S; Los Angeles Latino Eye Study Group. Psychometric performance of the NEI VFQ-25 in visually normal Latinos: the Los Angeles Latino Eye Study. Invest Ophthalmol Vis Sci. 2003 Apr;44(4):1470-8. PMID: 12657581

154. Ivers RQ, Cumming RG, Mitchell P, Simpson JM, Peduto AJ. Visual risk factors for hip fracture in older people. J Am Geriatr Soc. 2003 Mar;51(3):356-63. PMID: 12588579

155. Trautner C, Haastert B, Richter B, Berger M, Giani G. Incidence of blindness in southern Germany due to glaucoma and degenerative conditions. Invest Ophthalmol Vis Sci. 2003 Mar;44(3):1031-4. PMID: 12601025

156. Tsai SY, Cheng CY, Hsu WM, Su TP, Liu JH, Chou P. Association between visual impairment and depression in the elderly. J Formos Med Assoc. 2003 Feb;102(2):86-90. PMID: 12709736

157. Rotchford AP, Kirwan JF, Muller MA, Johnson GJ, Roux P. Temba glaucoma study: a population-based cross-sectional survey in urban South Africa. Ophthalmology. 2003 Feb;110(2):376-82. PMID: 12578784

158. Broman AT, Munoz B, Rodriguez J, Sanchez R, Quigley HA, Klein R, Snyder R, West SK. The impact of visual impairment and eye disease on vision-related quality of life in a Mexican-American population: proyecto VER. Invest Ophthalmol Vis Sci. 2002 Nov;43 (11):3393-8. PMID: 12407148

159. Mbulaiteye SM, Reeves BC, Karabalinde A, Ruberantwari A, Mulwanyi F, Whitworth JA, Johnson GJ. Evaluation of E-optotypes as a screening test and the prevalence and causes of visual loss in a rural population in SW Uganda. Ophthalmic Epidemiol. 2002 Oct;9(4):251-62. PMID: 12187423

160. Alagaratnam J, Sharma TK, Lim CS, Fleck BW. A survey of visual impairment in children attending the Royal Blind School, Edinburgh using the WHO childhood visual impairment database. Eye. 2002 Sep;16(5):557-61. PMID: 12194068

161. Thomas R, Parikh R, Paul P, Muliyil J. Population-based screening versus case detection.Indian J Ophthalmol. 2002 Sep;50(3):233-7. PMID: 12355704

162. Christian P. Recommendations for indicators: night blindness during pregnancy--a simple tool to assess vitamin A deficiency in a population. J Nutr. 2002 Sep;132(9 Suppl): 2884S-2888S. PMID: 12221265

163. Lupsakko T, Mantyjarvi M, Kautiainen H, Sulkava R. Combined hearing and visual impairment and depression in a population aged 75 years and older. Int J Geriatr Psychiatry. 2002 Sep;17(9):808-13. PMID: 12221653

164. Thiagalingam S, Cumming RG, Mitchell P. Factors associated with undercorrected refractive errors in an older population: the Blue Mountains Eye Study. Br J Ophthalmol. 2002 Sep;86(9):1041-5. PMID: 12185135

165. Narendran V, John RK, Raghuram A, Ravindran RD, Nirmalan PK, Thulasiraj RD. Diabetic retinopathy among self reported diabetics in southern India: a population based assessment. Br J Ophthalmol. 2002 Sep;86(9):1014-8. PMID: 12185129

166. Rahi J, Logan S, Timms C, Russell-Eggitt I, Taylor D. Risk, causes, and outcomes of visual impairment after loss of vision in the non-amblyopic eye: a population-based study. Lancet. 2002 Aug 24;360(9333):597-602. PMID: 12241931

167. Clarke P, Gray A, Holman R. Estimating utility values for health states of type 2 diabetic patients using the EQ-5D (UKPDS 62). Med Decis Making. 2002 Jul-Aug;22(4):340-9. PMID: 12150599

168. Dandona R, Dandona L, Kovai V, Giridhar P, Prasad MN, Srinivas M. Population-based study of spectacles use in southern India. Indian J Ophthalmol. 2002 Jun;50(2):145-55. PMID: 12194576

169. Mukesh BN, McCarty CA, Rait JL, Taylor HR. Five-year incidence of open-angle glaucoma: the visual impairment project. Ophthalmology. 2002 Jun;109(6):1047-51. PMID: 12045042

170. Azen SP, Varma R, Preston-Martin S, Ying-Lai M, Globe D, Hahn S. Binocular visual acuity summation and inhibition in an ocular epidemiological study: the Los Angeles Latino Eye Study. Invest Ophthalmol Vis Sci. 2002 Jun;43(6):1742-8. PMID: 12036974

171. Rotchford AP, Johnson GJ. Glaucoma in Zulus: a population-based cross-sectional survey in a rural district in South Africa. Arch Ophthalmol. 2002 Apr;120(4):471-8. PMID: 11934321

172. Vongphanit J, Mitchell P, Wang JJ. Prevalence and progression of myopic retinopathy in an older population. Ophthalmology. 2002 Apr;109(4):704-11. PMID: 11927427

173. Bertoni AG, Krop JS, Anderson GF, Brancati FL. Diabetes-related morbidity and mortality in a national sample of U.S. elders. Diabetes Care. 2002 Mar;25(3):471-5. PMID: 11874932

174. Bowman RJ, Faal H, Dolin P, Johnson GJ. Non-trachomatous corneal opacities in the Gambia--aetiology and visual burden. Eye. 2002 Jan;16(1):27-32. PMID: 11915876

175. Massof RW. A model of the prevalence and incidence of low vision and blindness among adults in the U.S. Optom Vis Sci. 2002 Jan;79(1):31-8. PMID: 11828896

176. Lau J, Michon JJ, Chan WS, Ellwein LB. Visual acuity and quality of life outcomes in cataract surgery patients in Hong Kong. Br J Ophthalmol. 2002 Jan;86(1):12-7. PMID: 11801495

177. Globe DR, Schoua-Glusberg A, Paz S, Yu E, Preston-Martin S, Azen S, Varma R. Using focus groups to develop a culturally sensitive methodology for epidemiological surveys in a Latino population: findings from the Los Angeles Latino Eye Study (LALES). Ethn Dis. 2002 Spring;12(2):259-66. PMID: 12019936

178. Van DE, Kulier R, Gulmezoglu AM, Villar J. Vitamin A supplementation during pregnancy. Cochrane Database Syst Rev. 2002;(4):CD001996. Review. PMID: 12519564

179. Lewallen S, Courtright P. Gender and use of cataract surgical services in developing countries. Bull World Health Organ. 2002;80(4):300-3. Review. PMID: 12075366

180. la Cour M, Kiilgaard JF, Nissen MH. Age-related macular degeneration: epidemiology and optimal treatment. Drugs Aging. 2002;19(2):101-33. Review. PMID: 11950377

181. Quigley HA, West SK, Rodriguez J, Munoz B, Klein R, Snyder R. The prevalence of glaucoma in a population-based study of Hispanic subjects: Proyecto VER. Arch Ophthalmol. 2001 Dec;119(12):1819-26. PMID: 11735794

182. Weih LM, Nanjan M, McCarty CA, Taylor HR. Prevalence and predictors of open-angle glaucoma: results from the visual impairment project. Ophthalmology. 2001 Nov;108 (11):1966-72. PMID: 11713063

183. Maini R, Keeffe J, Weih LA, McCarty CA, Taylor HR. Correction of refractive error in the Victorian population: the feasibility of "off the shelf" spectacles. Br J Ophthalmol. 2001 Nov;85(11):1283-6. PMID: 11673288

184. Prasad S, Kamath GG, Jones K, Clearkin LG, Phillips RP. Prevalence of blindness and visual impairment in a population of people with diabetes. Eye. 2001 Oct;15(Pt 5):640-3. PMID: 11702977

185. Sil AK, Gilbert C. Childhood blindness in India. J Indian Med Assoc. 2001 Oct;99 (10):557-60. PMID: 12018540

186. Rabiu MM, Abiose A. Magnitude of trachoma and barriers to uptake of lid surgery in a rural community of northern Nigeria. Ophthalmic Epidemiol. 2001 Jul;8(2-3):181-90. PMID: 11471087

187. Rabiu MM. Cataract blindness and barriers to uptake of cataract surgery in a rural community of northern Nigeria. Br J Ophthalmol. 2001 Jul;85(7):776-80. PMID: 11423446

188. Hameed TK, Hodge WG, Buhrmann R. An inventory of information on blindness and visual impairment in Canada. Can J Ophthalmol. 2001 Jun;36(4):175-85; discussion 185-6. PMID: 11428526

189. Courtright P, Lewallen S, Tungpakorn N, Cho BH, Lim YK, Lee HJ, Kim SH. Cataract in leprosy patients: cataract surgical coverage, barriers to acceptance of surgery, and outcome of surgery in a population based survey in Korea. Br J Ophthalmol. 2001 Jun;85(6):643-7. PMID: 11371479

190. Salvarani C, Hunder GG. Giant cell arteritis with low erythrocyte sedimentation rate: frequency of occurence in a population-based study. Arthritis Rheum. 2001 Apr;45(2):140-5. PMID: 11324777

191. McCarty CA, Nanjan MB, Taylor HR. Vision impairment predicts 5 year mortality. Br J Ophthalmol. 2001 Mar;85(3):322-6. PMID: 11222339

192. Al-Aqtum MT, Al-Qawasmeh MH. Prevalence of colour blindness in young Jordanians. Ophthalmologica. 2001 Jan-Feb;215(1):39-42. PMID: 11125268

193. Rubin GS, Bandeen-Roche K, Huang GH, Munoz B, Schein OD, Fried LP, West SK. The association of multiple visual impairments with self-reported visual disability: SEE project. Invest Ophthalmol Vis Sci. 2001 Jan;42(1):64-72. PMID: 11133849

194. Brown S, Weih L, Mukesh N, McCarty C, Taylor H. Assessment of adult stereopsis using the Lang 1 Stereotest: a pilot study. Binocul Vis Strabismus Q. 2001 summer;16 (2):91-8. PMID: 11388881

195. Dandona R, Dandona L, John RK, McCarty CA, Rao GN. Awareness of eye diseases in an urban population in southern India. Bull World Health Organ. 2001;79(2):96-102. Epub 2003 Sep 18. PMID: 11242828

196. Verma L, Das T, Binder S, Heriot WJ, Kirchhof B, Venkatesh P, Krebs I, Stolba U, Jahn C, Feichtinger H, Kellner L, Krugluger H, Pawelka I, Frohner U, Kruger A, Li W, Tewari HK. New approaches in the management of choroidal neovascular membrane in age-related macular degeneration. Indian J Ophthalmol. 2000 Dec;48(4):263-78. Review. PMID: 11340884

197. Schemann JF, Bakayoko S, Coulibaly S. Traditional couching is not an effective alternative procedure for cataract surgery in Mali. Ophthalmic Epidemiol. 2000 Dec;7(4):271-83. PMID: 11262674

198. Brown SA, Weih LM, Fu CL, Dimitrov P, Taylor HR, McCarty CA. Prevalence of amblyopia and associated refractive errors in an adult population in Victoria, Australia. Ophthalmic Epidemiol. 2000 Dec;7(4):249-58. PMID: 11262672

199. VanNewkirk MR, Weih L, McCarty CA, Stanislavsky YL, Keeffe JE, Taylor HR. Visual impairment and eye diseases in elderly institutionalized Australians. Ophthalmology. 2000 Dec;107(12):2203-8. PMID: 11097596

200. Rubin GS, Munoz B, Bandeen-Roche K, West SK. Monocular versus binocular visual acuity as measures of vision impairment and predictors of visual disability. Invest Ophthalmol Vis Sci. 2000 Oct;41(11):3327-34. PMID: 11006221

201. Dandona L, Dandona R, Srinivas M, John RK, McCarty CA, Rao GN. Ocular trauma in an urban population in southern India: the Andhra Pradesh Eye Disease Study. Clin Experiment Ophthalmol. 2000 Oct;28(5):350-6. PMID: 11097281

202. Dandona L, Dandona R, Mandal P, Srinivas M, John RK, McCarty CA, Rao GN. Angle-closure glaucoma in an urban population in southern India. The Andhra Pradesh eye disease study. Ophthalmology. 2000 Sep;107(9):1710-6. PMID: 10964834

203. Eloff J, Foster A. Cataract surgical coverage: results of a population-based survey at Nkhoma, Malawi. Ophthalmic Epidemiol. 2000 Sep;7(3):219-21. PMID: 11035556

204. Cheng CY, Liu JH, Chen SJ, Lee FL. Population-based study on prevalence and risk factors of age-related cataracts in Peitou, Taiwan. Zhonghua Yi Xue Za Zhi (Taipei). 2000 Aug;63(8):641-8. PMID: 10969451

205. VanNewkirk MR, Nanjan MB, Wang JJ, Mitchell P, Taylor HR, McCarty CA. The prevalence of age-related maculopathy: the visual impairment project. Ophthalmology. 2000 Aug;107(8):1593-600. PMID: 10919916

206. McKay R, McCarty CA, Taylor HR. Diabetic retinopathy in Victoria, Australia: the Visual Impairment Project. Br J Ophthalmol. 2000 Aug;84(8):865-70. PMID: 10906093

207. Dandona L, Dandona R, John RK, McCarty CA, Rao GN. Population based assessment of uveitis in an urban population in southern India. Br J Ophthalmol. 2000 Jul;84(7):706-9. PMID: 10873978

208. Taylor DJ, Jacob JS, Tooke JE. The integration of digital camera derived images with a computer based diabetes register for use in retinal screening. Comput Methods Programs Biomed. 2000 Jul;62(3):157-63. PMID: 10837903

209. Rochtchina E, Mitchell P. Projected number of Australians with glaucoma in 2000 and 2030. Clin Experiment Ophthalmol. 2000 Jun;28(3):146-8. PMID: 10981783

210. Dandona R, Dandona L, McCarty CA, Rao GN. Adaptation of WHOQOL as health-related quality of life instrument to develop a vision-specific instrument. Indian J Ophthalmol. 2000 Mar;48(1):65-70. PMID: 11271942

211. Hillen T, Lun A, Reischies FM, Borchelt M, Steinhagen-Thiessen E, Schaub RT. DHEA-S plasma levels and incidence of Alzheimer's disease. Biol Psychiatry. 2000 Jan 15;47(2):161-3. PMID: 10664834

212. Dandona R, Dandona L, Naduvilath TJ, McCarty CA, Rao GN. Utilisation of eyecare services in an urban population in southern India: the Andhra Pradesh eye disease study. Br J Ophthalmol. 2000 Jan;84(1):22-7. PMID: 10611094

213. Hofer TP, Vijan S, Hayward RA. Estimating the microvascular benefits of screening for type 2 diabetes mellitus. Int J Technol Assess Health Care. 2000 Summer;16(3):822-33. PMID: 11028137

214. Taylor HR, McCarty CA, Nanjan MB. Vision impairment predicts five-year mortality. Trans Am Ophthalmol Soc. 2000;98:91-6; discussion 96-9. PMID: 11190044

215. Monestam E, Wachtmeister L. Impact of cataract surgery on visual acuity and subjective functional outcomes: a population-based study in Sweden. Eye. 1999 Dec;13 (Pt 6):711-9. PMID: 10707130

216. Sreenivas V, Prabhakar AK, Badrinath SS, Fernandez T, Roy IS, Sharma T, Shah B. A rural population based case-control study of senile cataract in India. J Epidemiol. 1999 Nov;9(5):327-36. PMID: 10616266

217. Klein R, Klein BE, Moss SE, Cruickshanks KJ. Association of ocular disease and mortality in a diabetic population. Arch Ophthalmol. 1999 Nov;117(11):1487-95. PMID: 10565517

218. Dandona R, Dandona L, Naduvilath TJ, Srinivas M, McCarty CA, Rao GN. Refractive errors in an urban population in Southern India: the Andhra Pradesh Eye Disease Study. Invest Ophthalmol Vis Sci. 1999 Nov;40(12):2810-8. PMID: 10549640

219. Dandona L, Dandona R, Naduvilath TJ, McCarty CA, Rao GN. Population based assessment of diabetic retinopathy in an urban population in southern India. Br J Ophthalmol. 1999 Aug;83(8):937-40. PMID: 10413697

220. Clemett R, Darlow B. Results of screening low-birth-weight infants for retinopathy of prematurity. Curr Opin Ophthalmol. 1999 Jun;10(3):155-63. Review. PMID: 10537772

221. Dandona L, Dandona R, Naduvilath TJ, McCarty CA, Mandal P, Srinivas M, Nanda A, Rao GN. Population-based assessment of the outcome of cataract surgery in an urban population in southern India. Am J Ophthalmol. 1999 Jun;127(6):650-8. PMID: 10372874

222. Orr P, Barron Y, Schein OD, Rubin GS, West SK. Eye care utilization by older americans: the SEE Project. Salisbury Eye Evaluation. Ophthalmology. 1999 May; 106(5):904-9. PMID: 10328388

223. Wensor M, McCarty CA, Taylor HR. Prevalence and risk factors of myopia in Victoria, Australia. Arch Ophthalmol. 1999 May;117(5):658-63. PMID: 10326965

224. Klein BE, Klein R, Lee KE, Cruickshanks KJ. Associations of performance-based and self-reported measures of visual function. The Beaver Dam Eye Study. Ophthalmic Epidemiol. 1999 Mar;6(1):49-60. PMID: 10384684

225. Pukkala E, Verkasalo PK, Ojamo M, Rudanko SL. Visual impairment and cancer: a population-based cohort study in Finland. Cancer Causes Control. 1999 Feb;10(1):13-20. PMID: 10334637

226. Valbuena M, Bandeen-Roche K, Rubin GS, Munoz B, West SK. Self-reported assessment of visual function in a population-based study: the SEE project. Salisbury Eye Evaluation. Invest Ophthalmol Vis Sci. 1999 Feb;40(2):280-8. PMID: 9950585

227. Holmstrom G, el Azazi M, Kugelberg U. Ophthalmological follow up of preterm infants: a population based, prospective study of visual acuity and strabismus. Br J Ophthalmol. 1999 Feb;83(2):143-50. PMID: 10396188

228. Munoz B, West S, Rubin GS, Schein OD, Fried LP, Bandeen-Roche K. Who participates in population based studies of visual impairment? The Salisbury Eye Evaluation project experience. Ann Epidemiol. 1999 Jan;9(1):53-9. PMID: 9915609

229. McCarty CA, Keeffe JE, Taylor HR. The need for cataract surgery: projections based on lens opacity, visual acuity, and personal concern. Br J Ophthalmol. 1999 Jan;83(1):62-5. PMID: 10209437

230. Krumpaszky HG, Dietz K, Mickler A, Selbmann HK. Mortality in blind subjects. A population-based study on social security files from Baden-Wurttemberg. Ophthalmologica. 1999;213(1):48-53. PMID: 9838257

231. Morgan D, Jones C, Whitworth J, Ross A, Johnson G. Ocular findings in HIV-1 positive and HIV-1 negative participants in a rural population-based cohort in Uganda. Int Ophthalmol. 1998-99;22(3):183-92. PMID: 10548465

232. Herman WH, Aubert RE, Engelgau MM, Thompson TJ, Ali MA, Sous ES, Hegazy M, Badran A, Kenny SJ, Gunter EW, Malarcher AM, Brechner RJ, Wetterhall SF, DeStefano F, Smith PJ, Habib M, abd el Shakour S, Ibrahim AS, el Behairy EM. Diabetes mellitus in Egypt: glycaemic control and microvascular and neuropathic complications. Diabet Med. 1998 Dec;15(12):1045-51. PMID: 9868980

233. Jacobson L, Lundin S, Flodmark O, Ellstrom KG. Periventricular leukomalacia causes visual impairment in preterm children. A study on the aetiologies of visual impairment in a population-based group of preterm children born 1989-95 in the county of Varmland, Sweden. Acta Ophthalmol Scand. 1998 Oct;76(5):593-8. PMID: 9826046

234. Okongo M, Morgan D, Mayanja B, Ross A, Whitworth J. Causes of death in a rural, population-based human immunodeficiency virus type 1 (HIV-1) natural history cohort in Uganda. Int J Epidemiol. 1998 Aug;27(4):698-702. PMID: 9758128

235. Livingston PM, McCarty CA, Taylor HR. Knowledge, attitudes, and self care practices associated with age related eye disease in Australia. Br J Ophthalmol. 1998 Jul;82 (7):780-5. PMID: 9924371

236. Klaver CC, Kliffen M, van Duijn CM, Hofman A, Cruts M, Grobbee DE, van Broeckhoven C, de Jong PT. Genetic association of apolipoprotein E with age-related macular degeneration. Am J Hum Genet. 1998 Jul;63(1):200-6. Erratum in: Am J Hum Genet 1998 Oct;63(4):1252. PMID: 9634502

237. Van Newkirk MR, McCarty CA, Martone JF, Lam CS, Taylor HR. Methods for the Hong Kong Vision Study: a pilot assessment of visual impairment in adults. Ophthalmic Epidemiol. 1998 Jun;5(2):57-67. PMID: 9672906

238. Pokharel GP, Selvaraj S, Ellwein LB. Visual functioning and quality of life outcomes among cataract operated and unoperated blind populations in Nepal. Br J Ophthalmol. 1998 Jun;82(6):606-10. PMID: 9797658

239. McCarty CA, Bansal AK, Livingston PM, Stanislavsky YL, Taylor HR. The epidemiology of dry eye in Melbourne, Australia. Ophthalmology. 1998 Jun;105 (6):1114-9. PMID: 9627665

240. Moss SE, Klein R, Klein BE. The 14-year incidence of visual loss in a diabetic population. Ophthalmology. 1998 Jun;105(6):998-1003. PMID: 9627648

241. Pieramici DJ, Bressler SB. Age-related macular degeneration and risk factors for the development of choroidal neovascularization in the fellow eye. Curr Opin Ophthalmol. 1998 Jun;9(3):38-46. Review. PMID: 10182098

242. Ek U, Fernell E, Jacobson L, Gillberg C. Relation between blindness due to retinopathy of prematurity and autistic spectrum disorders: a population-based study. Dev Med Child Neurol. 1998 May;40(5):297-301. PMID: 9630256

243. Sumich P, Mitchell P, Wang JJ. Choroidal nevi in a white population: the Blue Mountains Eye Study. Arch Ophthalmol. 1998 May;116(5):645-50. PMID: 9596501

244. McCarty CA, Lloyd-Smith CW, Lee SE, Livingston PM, Stanislavsky YL, Taylor HR. Use of eye care services by people with diabetes: the Melbourne Visual Impairment Project. Br J Ophthalmol. 1998 Apr;82(4):410-4. Erratum in: Br J Ophthalmol 1998 May;82(5):591. PMID: 9640191

245. Christian P, West KP Jr, Khatry SK, Katz J, Shrestha SR, Pradhan EK, LeClerq SC, Pokhrel RP. Night blindness of pregnancy in rural Nepal--nutritional and health risks. Int J Epidemiol. 1998 Apr;27(2):231-7. PMID: 9602403

246. Wensor MD, McCarty CA, Stanislavsky YL, Livingston PM, Taylor HR. The prevalence of glaucoma in the Melbourne Visual Impairment Project. Ophthalmology. 1998 Apr;105 (4):733-9. PMID: 9544649

247. Jacobson L, Fernell E, Broberger U, Ek U, Gillberg C. Children with blindness due to retinopathy of prematurity: a population-based study. Perinatal data, neurological and ophthalmological outcome. Dev Med Child Neurol. 1998 Mar;40(3):155-9. PMID: 9566650

248. Sauve RS, Robertson C, Etches P, Byrne PJ, Dayer-Zamora V. Before viability: a geographically based outcome study of infants weighing 500 grams or less at birth. Pediatrics. 1998 Mar;101(3 Pt 1):438-45. PMID: 9481011

249. Attebo K, Mitchell P, Cumming R, Smith W, Jolly N, Sparkes R. Prevalence and causes of amblyopia in an adult population. Ophthalmology. 1998 Jan;105(1):154-9. PMID: 9442792

250. Zerihun N. Trachoma in Jimma zone, south western Ethiopia. Trop Med Int Health. 1997 Dec;2(12):1115-21. PMID: 9438465

251. Trevathan E, Murphy CC, Yeargin-Allsopp M. Prevalence and descriptive epidemiology of Lennox-Gastaut syndrome among Atlanta children. Epilepsia. 1997 Dec;38(12):1283-8. PMID: 9578523

252. Dandona R, Dandona L, Naduvilath TJ, Nanda A, McCarty CA. Design of a population-based study of visual impairment in India: The Andhra Pradesh Eye Disease Study. Indian J Ophthalmol. 1997 Dec;45(4):251-7. Review. PMID: 9567024

253. Courtright P, Kim SH, Lee HS, Lewallen S. Excess mortality associated with blindness in leprosy patients in Korea. Lepr Rev. 1997 Dec;68(4):326-30. PMID: 9503868

254. Reischies FM, Geiselmann B. Age-related cognitive decline and vision impairment affecting the detection of dementia syndrome in old age. Br J Psychiatry. 1997 Nov; 171:449-51. PMID: 9463604

255. Clendenin C, Coffey M, Marsh M, West S. Eye care utilisation patterns in a rural county in Ireland: implications for service delivery. Br J Ophthalmol. 1997 Nov;81(11):972-5. PMID: 9505821

256. Attebo K, Mitchell P, Cumming R, Smith W. Knowledge and beliefs about common eye diseases. Aust N Z J Ophthalmol. 1997 Nov;25(4):283-7. PMID: 9395831

257. Monestam E, Wachtmeister L. The impact of cataract surgery on low vision patients. A population based study. Acta Ophthalmol Scand. 1997 Oct;75(5):569-76. PMID: 9469558

258. Frederiksen JL, Sorensen TL, Sellebjerg FT. Residual symptoms and signs after untreated acute optic neuritis. A one-year follow-up. Acta Ophthalmol Scand. 1997 Oct;75(5):544-7. PMID: 9469554

259. Shah B, Prabhakar AK. Chronic morbidity profile among elderly. Indian J Med Res. 1997 Oct;106:265-72. Review. PMID: 9361460

260. Jingjing X, Wenshu M, Shaozhen L, Lezheng W, Zhongyao W, Siping Z, Qiang Y, Mingguang H. A model for blindness prevention. Yan Ke Xue Bao. 1997 Sep;13(3):162-3, 147. PMID: 11326870

261. Livingston PM, McCarty CA, Taylor HR. Visual impairment and socioeconomic factors. Br J Ophthalmol. 1997 Jul;81(7):574-7. PMID: 9290372

262. Icks A, Trautner C, Haastert B, Berger M, Giani G. Blindness due to diabetes: population -based age- and sex-specific incidence rates. Diabet Med. 1997 Jul;14(7):571-5. PMID: 9223395

263. Trautner C, Icks A, Haastert B, Plum F, Berger M. Incidence of blindness in relation to diabetes. A population-based study. Diabetes Care. 1997 Jul;20(7):1147-53. PMID: 9203453

264. Livingston PM, Lee SE, McCarty CA, Taylor HR. A comparison of participants with non-participants in a population-based epidemiologic study: the Melbourne Visual Impairment Project. Ophthalmic Epidemiol. 1997 Jun;4(2):73-81. PMID: 9243651

265. McCarty CA, Livingston PM, Taylor HR. Prevalence of myopia in adults: implications for refractive surgeons. J Refract Surg. 1997 May-Jun;13(3):229-34. Review. PMID: 9183754

266. Hirvela H, Laatikainen L. Diabetic retinopathy in people aged 70 years or older. The Oulu Eye Study. Br J Ophthalmol. 1997 Mar;81(3):214-7. PMID: 9135385

267. Rubin GS, West SK, Munoz B, Bandeen-Roche K, Zeger S, Schein O, Fried LP. A comprehensive assessment of visual impairment in a population of older Americans. The SEE Study. Salisbury Eye Evaluation Project. Invest Ophthalmol Vis Sci. 1997 Mar; 38(3):557-68. PMID: 9071208

268. Erie JC, Hodge DO, Gray DT. The incidence of primary angle-closure glaucoma in Olmsted County, Minnesota. Arch Ophthalmol. 1997 Feb;115(2):177-81. PMID: 9046251

269. Mitchell P, Hayes P, Wang JJ. Visual impairment in nursing home residents: the Blue Mountains Eye Study. Med J Aust. 1997 Jan 20;166(2):73-6. PMID: 9033561

270. Kristinsson JK. Diabetic retinopathy. Screening and prevention of blindness. A doctoral thesis. Acta Ophthalmol Scand Suppl. 1997;(223):1-76. PMID: 9559048

271. Sparrow JM, Dickinson AJ, Duke AM, Thompson JR, Gibson JM, Rosenthal AR. Seven year follow-up of age-related maculopathy in an elderly British population. Eye. 1997;11 (Pt 3):315-24. PMID: 9373468

272. Dickinson AJ, Sparrow JM, Duke AM, Thompson JR, Gibson JM, Rosenthal AR. Prevalence of age-related maculopathy at two points in time in an elderly British population. Eye. 1997;11 (Pt 3):301-14. PMID: 9373467

273. Schaumberg DA, O'Connor J, Semba RD. Risk factors for xerophthalmia in the Republic of Kiribati. Eur J Clin Nutr. 1996 Nov;50(11):761-4. PMID: 8933124

274. Foster PJ, Baasanhu J, Alsbirk PH, Munkhbayar D, Uranchimeg D, Johnson GJ. Glaucoma in Mongolia. A population-based survey in Hovsgol province, northern Mongolia. Arch Ophthalmol. 1996 Oct;114(10):1235-41. PMID: 8859083

275. Steffenburg U, Hagberg G, Kyllerman M. Characteristics of seizures in a population-based series of mentally retarded children with active epilepsy. Epilepsia. 1996 Sep;37(9):850-6. PMID: 8814097

276. Hagberg B, Hagberg G, Olow I, van Wendt L. The changing panorama of cerebral palsy in Sweden. VII. Prevalence and origin in the birth year period 1987-90. Acta Paediatr. 1996 Aug;85(8):954-60. PMID: 8863878

277. Desai P, MacEwen CJ, Baines P, Minassian DC. Incidence of cases of ocular trauma admitted to hospital and incidence of blinding outcome. Br J Ophthalmol. 1996 Jul; 80(7):592-6. PMID: 8795369

278. Nodgaard H, Andreasen H, Hansen H, Sorensen HT. Risk factors associated with retinopathy of prematurity (ROP) in northern Jutland, Denmark 1990-1993. Acta Ophthalmol Scand. 1996 Jun;74(3):306-10. PMID: 8828733

279. Evans J, Wormald R. Is the incidence of registrable age-related macular degeneration increasing? Br J Ophthalmol. 1996 Jan;80(1):9-14. PMID: 8664242

280. Rosenberg T, Flage T, Hansen E, Riise R, Rudanko SL, Viggosson G, Tornqvist K. Incidence of registered visual impairment in the Nordic child population. Br J Ophthalmol. 1996 Jan;80(1):49-53. PMID: 8664232

281. Javitt JC, Aiello LP. Cost-effectiveness of detecting and treating diabetic retinopathy. Ann Intern Med. 1996 Jan 1;124(1 Pt 2):164-9. PMID: 8554212

282. McCarty CA, Lee SE, Livingston PM, Bissinella M, Taylor HR. Ocular exposure to UV-B in sunlight: the Melbourne visual impairment project model. Bull World Health Organ. 1996;74(4):353-60. PMID: 8823956

283. Modarres M, Mirsamadi M, Peyman GA. Prevalence of congenital color deficiencies in secondary-school students in Tehran. Int Ophthalmol. 1996-97;20(4):221-2. PMID: 9112190
